# Disentangling the determinants of species richness of vascular plants and mammals from national to regional scales

**DOI:** 10.1038/srep21988

**Published:** 2016-02-23

**Authors:** Haigen Xu, Mingchang Cao, Yi Wu, Lei Cai, Yun Cao, Jun Wu, Juncheng Lei, Zhifang Le, Hui Ding, Peng Cui

**Affiliations:** 1 Nanjing Institute of Environmental Sciences, Ministry of Environmental Protection, Nanjing 210042, China; 2 College of Forest Resources and Environment, Nanjing Forestry University, Nanjing 210037, China; 3 Department of Natural Ecology Conservation, Ministry of Environmental Protection, Beijing 100035, China; 4 Department of Biology, Nanjing University, Nanjing 210093, China

## Abstract

Understanding the spatial patterns in species richness gets new implication for biodiversity conservation in the context of climate change and intensified human intervention. Here, we created a database of the geographical distribution of 30,519 vascular plant species and 565 mammal species from 2,376 counties across China and disentangled the determinants that explain species richness patterns both at national and regional scales using spatial linear models. We found that the determinants of species richness patterns varied among regions: elevational range was the most powerful predictor for the species richness of plants and mammals across China. However, species richness patterns in the Qinghai-Tibetan Plateau Region (QTR) are quite unique, where net primary productivity was the most important predictor. We also detected that elevational range was positively related to plant species richness when it is less than 1,900 m, whereas the relationship was not significant when elevational range is larger than 1,900 m. It indicated that elevational range often emerges as the predominant controlling factor within the regions where energy is sufficient. The effects of land use on mammal species richness should attract special attention. Our study suggests that region-specific conservation policies should be developed based on the regional features of species richness.

Biodiversity is distributed heterogeneously over the globe^1^. Species richness, as one of the major surrogates of biodiversity, reaches the peak around the equator where it is warm and wet, and experiences a decline toward the temperate and polar regions where it is colder and drier^2^. For more than two centuries, ecologists and biogeographers have been exploring the mechanism that underpins species richness patterns of diverse biological taxa across the earth^3^,^4^,^5^,^6^,^7^,^8^. But there has always been no consensus on this issue. As the climate change and human intervention have already exerted great influences on species richness patterns and even triggered species extinction^9^,^10^,^11^,^12^,^13^, this issue gets new implication for biodiversity conservation in this century. Therefore, more intensive and in-depth researches are needed to explore the mechanism of species richness patterns.

Spatial patterns of species richness are the complex product created by the interaction of a series of biotic and abiotic factors^7^,^14^. Biotic factors are biological intrinsic characters, e.g. anatomy, physiology, genetics, development and behavior^14^. Abiotic factors often encompass climate, topography, geographical history^15^,^16^,^17^,^18^, etc. To date, a plethora of hypotheses have been proposed to identify the environmental factors that explain patterns of species richness. Among them, such hypotheses have received the strongest empirical support, e.g. the energy hypothesis, the environmental stability hypothesis and the habitat heterogeneity hypothesis^16^,^19^,^20^,^21^,^22^,^23^,^24^. The energy hypothesis posits that water-energy dynamics, ambient energy, and productivity are responsible for species richness gradients^16^,^25^,^26^. The environmental stability hypothesis insists that a stable environment could house more species by accelerating species specialization and ecological niche diversification^22^,^26^. The habitat heterogeneity hypothesis states that heterogeneity in habitats is responsible for geographical variation in species richness^24^ because variability in elevation, landscape or vegetation could make diverse habitats for more species^26^. Nevertheless, the validity of these hypotheses remains controversial and the environmental factors that predominantly shape species richness patterns require more rigorous verification.

China is one of the countries with the richest biodiversity^27^,^28^. It harbors more than 30,000 vascular plant species and 6,300 vertebrate species respectively^27^,^29^, accounting for over 10% of the total number of the world^30^. As a result of obvious disparity in climatological, geographical and topographical features, China is generally categorized into three major regions: the Eastern Monsoon Region (EMR), the Northwestern Arid Region (NAR) and the Qinghai-Tibetan Plateau Region (QTR)^31^ (Fig. 1). Compared to EMR, species in NAR and QTR show a higher level of endemism and more sensitivity to the climate change^32^,^33^,^34^,^35^,^36^, which offers a good opportunity to disentangle the environmental determinants of species richness and get insight into the keystone of biodiversity conservation. Previous studies have made advancement in species richness of one or several taxa in a single region across China^32^,^33^,^34^,^35^,^37^,^38^,^39^,^40^. However, a comprehensive study of species richness and its determinants is scarce both at national and regional scales for effective biodiversity conservation in China.

In this study, we created a database of the geographical distribution of 30,519 vascular plant species and 565 mammal species from 2,376 counties in the terrestrial and inland water ecosystems of China, with objectives to disentangle the determinants of species richness of vascular plants and mammals across the whole country and in the three separate regions (EMR, NAR and QTR), and explore its implications for biodiversity conservation both at national and regional scales in China.

## Results

We found that species richness of vascular plants and mammals in China was higher in South China than in North China, and higher in the mountains than in the plains. As for the vascular plants, such regions harbor the highest species richness, i.e., the Min Mountains, the Qionglai Mountains, the Hengduan Mountains, the southeastern Himalaya Mountains, the Qinling Mountains, the Funiu Mountains, the Daba Mountains, the Dabie Mountains, the Wuling Mountains, the Wuyi Mountains, the Nanling Mountains, the Xishuangbanna in Yunnan Province, the mountains of southeastern Yunnan-western Guangxi-southern Guizhou, the mountains of southwestern Guangxi, the mountains of central and southern Hainan, and Taiwanese mountains (Fig. 2). All the hot spots covered 125 assessment units of 17 provinces (autonomous regions), among which, 116 assessment units are from EMR and 9 from QTR. Species richness of vascular plants reached the maximum (i.e., 3238) in Yulong, Yunnan. Species richness of vascular plants ranged from 1102 to 1533 in such regions, i.e., the hills in Zhejiang and Fujian, the hills in southern Anhui, the hills in Guangdong and Guangxi, the Changbai Mountains, and the Taihang Mountains. Species richness in most assessment units of other regions was less than 394, e.g. the Qinghai-Tibet Plateau, the Qaidam Basin, the Tarim Basin, the Inner Mongolian Plateau, the Northeast China Plain, the North China Plain, and the Chengdu Plain. As for the mammals, the distribution pattern of species richness can also be found in Xu *et al.*^41^. The hot spots included 49 assessment units of 10 provinces (autonomous regions).

We developed spatial linear models (SLM) for the species richness of vascular plants and mammals across China (Table 1). We identified elevational range was the most important predictor for vascular plants (z = 15.68, p < 0.001) and mammals (z = 9.59, p < 0.001). Water-energy variables (mean annual precipitation, temperature and net primary productivity), environmental stability (temperature annual range and precipitation seasonality) and main land cover type also played important roles in explaining the variance of the species richness of vascular plants and mammals across China (Table 1). These core predictors together explain 61% and 53% of the variance of the two taxa, respectively. When considering all 19 environmental variables, change in model fit was very small (Δr^2^ = 0.01) compared to SLM models with six variables (Table 1). Therefore, the best SLM models were robust.

We also established SLM models for species richness of vascular plants and mammals in EMR, NAR, and QTR, respectively. Changes in fitted values between SLM models with six predictors and those with 19 environmental variables were small (Δr^2^ ranges from 0.00 to 0.07) (Table 1). Therefore, these best SLM models were robust. Elevational range, the most important predictor of plant and mammal species richness across China, becomes the second or third important predictor of species richness of vascular plants and mammals in QTR. The value of z decreased from 15.68 across China to 4.15 in QTR for plant species, and from 9.59 across China to 3.79 in QTR for mammal species (Table 1). Net primary productivity became the most important factor affecting the distribution of plant and mammal species (z = 5.59 and 4.46, p < 0.001, respectively) in QTR.

We analyzed sampling bias in the dataset of this study. We identified 2010 counties that are ‘under-sampled’ based on the inventory incompleteness higher than 0.05^42^. Among the 2010 counties, the number of vascular plant species in the dataset of this study was higher in 1749 counties (87%), identical in two counties (approximately 0%) and less in 259 counties (13%) than that of Yang *et al.*^42^ (Fig. 3). The mean and standard deviation in the difference of vascular plant species richness between the two datasets were 305.2 and 314.3, respectively. Besides, we compared the number of vascular plant species per county between 217 counties in the dataset of this study and 217 nature reserves. As a result, we found that the number of vascular plant species in these counties and nature reserves were basically approximate (r = 0.92, P < 0.01, Fig. 4). From the above two aspects, it suggests that the accuracy of the data on species geographical distribution at county level was greatly improved and sampling bias can be reduced to a larger extent.

We found that the consistent results were obtained in GLM models based on different proportions of samples (60%, 70%, 80% and 90%) compared to that of multivariate models in target regions (100%) (Table S4). The top six environmental variables that reached statistical significance for the most times remained unchanged in GLM models based on the above sets of data (from 60% to 100%). For instance, as for vascular plants in whole China, the top six environmental variables were elevational range, net primary productivity, precipitation seasonality, mean annual precipitation, maximum temperature of the warmest month, and main land cover type. As for mammals in whole China, the top six environmental variables were elevational range, net primary productivity, main land cover type, maximum temperature of the warmest month, precipitation seasonality, and temperature annual range (Table S4).

We detected that the correlation coefficients between elevational range and some other environmental variables (i.e. precipitation, temperature and productivity) across China were less than 0.2 (Table S1). Similarly, the correlation coefficients between elevational range and other environmental variables in QTR were less than 0.3, except the minimum temperature of the coldest month and the number of main land types (Table S1). In addition, the effect of elevational range on plant species richness was not linear but marginal beyond certain threshold. As is shown in Fig. 5, elevational range was positively related to vascular plant species richness when elevational range is less than 1,900 m, whereas the relationship was marginal when elevational range is larger than 1,900 m. Areas with elevational range larger than 1,900 m are mostly low in annual potential evapotranspiration (less than 1,000 mm) and are mainly distributed in southwestern and northwestern part of China (Fig. 6).

## Discussion

According to the methods in the study of Jetz and Rahbek^43^ and Kreft & Jetz^24^, we assigned importance of some variables to species richness based on the z-score in SLM models. The higher z-score of a variable shows its more remarkable effect on species richness and the more important role of its relevant hypothesis. We found that elevational range was the most dominant factor of vascular plant species richness across China. Similar findings were reported in previous studies of vascular plants in the nature reserves of China^44^, the Jura Mountains of Switzerland^45^ and the Iberian Peninsula^46^. These similar findings, coupled with our results may be illustrated by the fact that all the above study sites are mostly composed of mountainous regions with relatively higher elevational range. Mountains provide a wide variety of habitats for species formation and specialization and buffering against climate change^47^,^48^. More microscopically, elevational transects in the mountains are nested within a biogeographic region and form a test system where the flora and fauna have gone through a similar geological and evolutionary history for the development of diverse species^49^. Kreft and Jetz^24^ identified potential evapotranspiration and the number of wet days per year as the two most important predictors of species richness of vascular plants across 1,032 geographic regions worldwide. The disparity between our conclusions may result from the different study regions and assessment units: the study of Kreft and Jetz^24^ was at global scale based on the assessment unit of about 12,100 km^2^ while our study was at national scale based on the assessment unit of about 3908.7 km^2^. Wang *et al.*^38^ stated that the mean temperature of the coldest quarter was the strongest predictor of species richness of woody plants in China. The different conclusions from our studies are probably due to the fact that spatial autocorrelation was not accounted for by GLM models in Wang *et al.*’s study, while our results were based on SLM models in which spatial autocorrelation was accounted for. Qian^40^ found that temperature seasonality was the best predictor of woody species richness in China based on the assessment unit of province, which is obviously larger than county, since the effect of habitat heterogeneity on species richness is stronger at fine scales than that at broad scales^50^,^51^.

In the three major regions of China, we detected that elevational range was not the most prominent predictor for the species richness of vascular plants in EMR. Our results demonstrated that elevational range was positively correlated with species richness when elevational range was less than 1,900 m, beyond which the relationship was not significant (Fig. 5). QTR fall within grey patches (elevational range is larger than 1,900 m; Fig. 6). The grey patches contain mostly low-energy regions where annual potential evapotranspiration is less than 1,000 mm. It revealed that in the regions with low availability of energy, species richness was often correlated with energy-related variables instead of elevational range. Conversely, elevational range often took the predominant role within the regions with sufficient energy for the survival and development of species. It is basically consistent with the previous studies in North America where mammals’ species richness is determined by habitat heterogeneity in the high-energy regions^6^. However, elevational range also greatly contributes to species richness of vascular plants and mammals (Table 2) and is slightly associated with most of other environmental variables both at national and regional scales (Table S1). It suggests that elevational range is a robust and relatively independent determinant at different scales. Zhao and Fang^44^ got the similar conclusion based on the study of vascular plants in nature reserves of the subtropical forest region, temperate forest region, temperate steppe and desert region, and the Qinghai-Tibet Plateau region. Since elevational range is vital to species richness, it suggests that habitat heterogeneity should be taken seriously in the work of biodiversity conservation.

Net primary productivity was considered as the most significant determinant for vascular plant species richness in QTR. The result was supported by the study of alpine meadow by Wang *et al.*^33^ in the same region. As the QTR and the Himalayas underwent accelerating uplift through the Quaternary, the interior became progressively desiccated as the influx of Indian Ocean moisture was constrained^28^. Thus, annual precipitation is low in most parts of QTR and unevenly distributed, e.g. 100–300 mm at the center of QTR and 3000 mm in the eastern part of QTR^52^. Besides, it is also characterized by low temperatures: the annual mean air temperature is −1.7 °C^53^. Accordingly, the richest plant species occur in the parts where the conditions of water and heat are sufficient for species formation and specialization. Hence, low temperature, little precipitation, and the strong variability make the productivity-related factors responsible for species richness patterns in the region and the biodiversity monitoring using the productivity-related indicators is urgently needed. Similarly, we found that normalized difference vegetation index (NDVI) was the second most prominent determinant of vascular plant species richness in NAR. It is basically consistent with the results of such previous studies of vascular plants in Kenya, Israel and USA^54^,^55^,^56^ since all the study regions harbor drier and colder environment. Li *et al.*^34^ identified that water availability was mostly correlated with plant species richness and supported the water-energy dynamics hypothesis. This result is similar to our study because NDVI and mean annual precipitation are highly correlated in NAR (r = 0.77, p < 0.01). Interestingly, some plants (e.g., *Lomatogoniopsis*) have been progressively evolving drought-tolerance or cold-adapted features, resulting in a drastic shift and uniqueness in the distribution of plant communities in the above regions. Thus, the endemic species’ ecological and evolutionary history should be highlighted in the making and implementation of conservation strategy^51^,^57^.

As for mammals, elevational range was also discovered as the most important predictor of species richness across China. Similar to vascular plants, determinants that dominantly shape the species richness pattern were also found to vary at different scales. At the regional scale, elevational range remains the most important predictor for mammals in EMR and NAR, however became the third important predictor in QTR. Mean annual precipitation was identified as the second most important determinants for mammals in EMR. It can be explained by precipitation gradients along latitude, with rich precipitation in the South where mammal species richness is high and low precipitation in the North where mammal species richness is low. Net primary productivity was the most important predictor for mammals in QTR. The reason is similar for plants in QTR. Net primary productivity is low in interior parts of QTR because the uplift of the Himalayas makes the interior become progressively desiccated^28^, resulting in low species richness there. Meanwhile, higher species richness of mammals occurs in the eastern and southeastern parts of QTR where net primary productivity is higher due to relatively sufficient precipitation and heat there (Fig. 2). As a result of the low ability of mammals to utilize the low water content forage in dry regions^58^, the efficiency of food chain is obviously lower than that with permanent water sources. The shortage of water and food makes the survival and specialization of mammals difficult in the extremely stressing status. Thus, mammal species richness is higher in the wetter regions than in the drier regions. The global climate change greatly influences the annual precipitation in QTR^59^,^60^ and tends to alter the species richness pattern accordingly, which should be particularly emphasized in the conservation of mammal species. In addition, precipitation seasonality was responsible for species richness in QTR. It is consistent with the previous study of a savannah large mammal community in the Amboseli ecosystem, Africa^58^. Both the study regions harbor the arid or semi-arid climate and show strong precipitation seasonality^52^. It is also discovered that main land cover type has emerged as the key factors to impact the mammal species richness in the whole country (z = −7.11) and EMR (z = −4.02) (Table 1). The result can be explicitly explained by the recent conspicuous changes in land use pattern across China, such as in the tropical mountains of Xishuangbanna, Yunnan and the farming-pasturing interlock region of northern China^61^,^62^. Hence, the effects of land use on mammal species richness should attract special attention.

Sampling bias may introduce errors into the results of our study. Two types of errors are possible, i.e., omission errors and commission errors. Here, we illustrated the sources of errors as follows: 1) Omission errors. The current dataset is much more prone to omission errors. Like most of the published databases, our database also has the ubiquitous shortcoming that sampling efforts are not uniform in space^63^,^64^,^65^,^66^. Most records of species distribution are derived from opportunistic collections without a unified sampling strategy to cover the full variation of environmental conditions in the entire target region. Sampling biases are common as records can be spatially biased towards more popular species or easily accessible regions^63^,^67^. Some species receive special attention, or are easy to be detected. Some regions are close to researchers, or have more research funding^68^,^69^. Some poorly known species and regions are most likely to be affected by the above limitations. As detailed surveys across the entire possible range are barely conducted due to lack of resources^70^,^71^, a lot of species that are actually present have not yet been recorded. As only a few counties in China have been surveyed with the aim of generating complete species lists, omission errors occur in the data on species distribution in some counties of China^72^. By contrast, well-surveyed regions are less likely to have omission errors. 2) Commission errors. Species may be misidentified or the locations may be wrongly recorded, which result in commission errors^63^,^69^. Species occurrence is not fixed especially for mammals as they move dynamically in time and space as a consequence of changing biotic and abiotic conditions. Due to change of habitats, such as transformation of forests into croplands, and wetlands into rice paddies, the distribution of species is likely to change. Thus, data on species distribution based on literatures or specimens may overestimate species distribution and then lead to commission errors in this analysis.

To reduce the sampling bias of species distribution data, we first organized more than 20 expert meetings and invited over 50 senior experts that are specialized in taxonomy and ecology of nearly all the specific taxa included in this study and rich experienced in field survey of vascular plants and mammals to review the data of spatial distribution of each species at the county level across China. Our invited experts carefully checked the list of species names. More importantly, they also comprehensively rectified the distribution information of each species. Through this process, the errors especially commission errors derived from sampling bias have been reduced as far as possible. In a second step, we made comparison of the inventory completeness of vascular plants between this study and the published study of Yang *et al.*^42^. Among the 2010 ‘under-sampled’ counties based on the definition of Yang *et al.*^42^, the inventory completeness in 87% of counties was greatly improved. Moreover, we compared the number of vascular plant species per county among 217 counties in the dataset of this study with species numbers that were compiled from the full-surveyed information from 217 nature reserves nested within the relevant counties. The result showed that the number of vascular plant species in these counties and nature reserves were basically approximate. Thus, we can comprehensively reduce sampling bias to a larger extent and improve the accuracy of the data on species geographical distribution at the county level.

To further test the impact of sampling bias on the robustness of the final (GLM) models, we performed a bootstrap procedure with stratified random sampling^73^,^74^,^75^,^76^. This method has been reasonably applied to explore the effects of sampling bias on the research result in the study of Muir *et al.*^77^. We selected subsets of samples (60%, 70%, 80% and 90%) from the target regions (i.e., whole China, EMR, NAR and QTR respectively) using stratified random sampling with a bootstrap procedure, and compared the multivariate models based on the subsets of samples with that of target regions (100%). When p < 0.05, the regression coefficient is considered statistically significant. This process was replicated 1000 times with randomly generated samples. We counted the number of times the regression coefficient of each variable reached statistical significance. The top six variables were selected according to the number of times each variable reached statistical significance among 1000 times. We found that the consistent results were obtained in GLM models based on different proportions of samples (from 60% to 90%), as compared with that of multivariate models in target regions (100%). The top six environmental variables that reached statistical significance for the most times remained unchanged in GLM models based on the above sets of data (from 60% to 100%) (Table S4). Therefore, we may basically conclude that the impact of sampling bias on multivariate models can be effectively controlled and the multivariate models are robust in this study. Though this method is still in the infant stage, we believe that it can offer a new way to test the effects of sampling bias.

In summary, our study provides insights into spatial patterns in species richness of vascular plants and mammals at national and regional scales. The relative contribution of variables that explain species richness patterns varied among regions. Elevational range was the most important predictor for plants and mammals across China. However, the uplift of the Himalayas makes spatial patterns of species richness in QTR quite unique, where net primary productivity was the most controlling factor for plants and mammals. Elevational range is often independent at different scales and emerges as the predominant controlling factor in the regions with sufficient energy for the survival and development of species. Therefore, we suggest that region-specific conservation policies should be developed based on the regional features of species richness.

## Methods

### Study area

**Figure 1:**
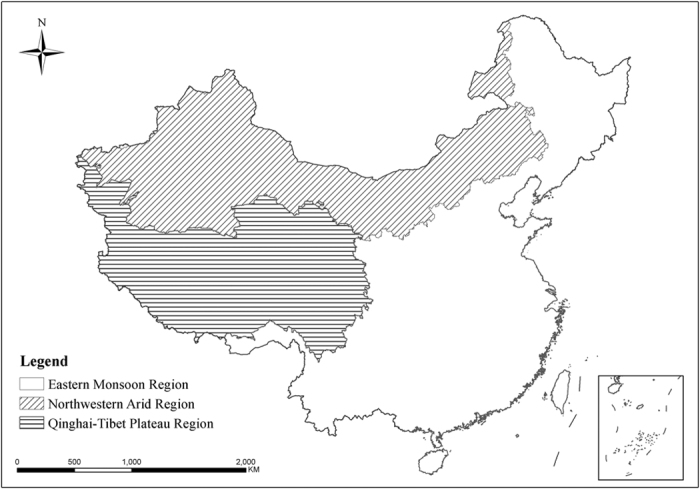
Illustrative map of the Eastern Monsoon Region (EMR), the Northwestern Arid Region (NAR), and the Qinghai-Tibetan Plateau Region (QTR) in the terrestrial and inland water ecosystems of China. The inset in the right bottom of the figure shows the southern boundary of China, including all islands in the South China Sea. Data on national territory were from the National Administration of Surveying, Mapping and Geoinformation of China (http://www.sbsm.gov.cn/). The map was created using the software Arc GIS 9.3 which was purchased from ESRI-China (http://www.esrichina-bj.cn/) by Nanjing Institute of Environmental Sciences affiliated to the Ministry of Environmental Protection of China, with user number C-801668.

**Figure 2:**
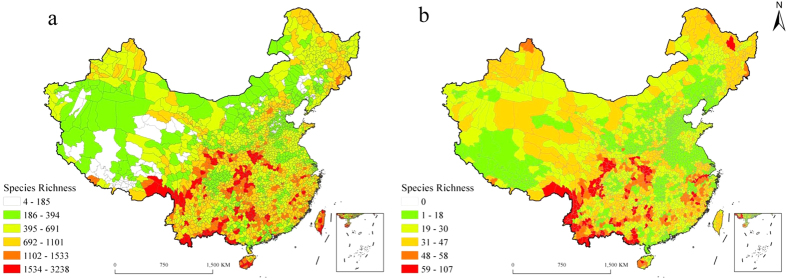
Spatial distribution map of vascular plants and mammals in China. (**a**) vascular plants; (**b**) mammals. Red areas are hotspots defined as the richest 5% of county areas for plant and mammal species. Data on the boundary of assessment units were from the National Administration of Surveying, Mapping and Geoinformation of China (http://www.sbsm.gov.cn/). The map was created using the software Arc GIS 9.3 which was purchased from ESRI-China (http://www.esrichina-bj.cn/) by Nanjing Institute of Environmental Sciences affiliated to the Ministry of Environmental Protection of China, with user number C-801668.

**Figure 3:**
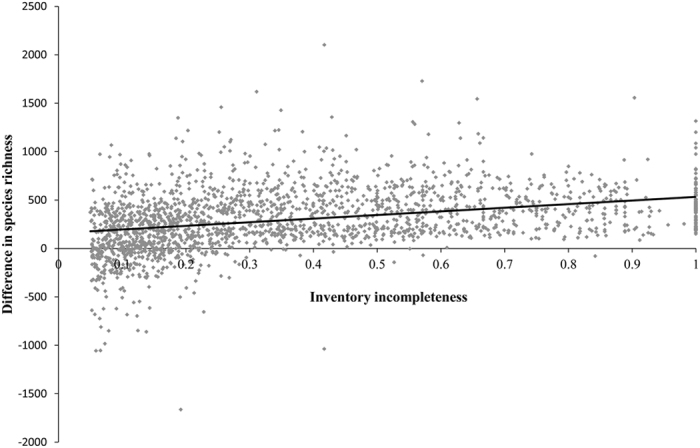
Difference in the number of vascular plant species between the dataset of this study and that of Yang *et al.* 42. X-axis is the inventory incompleteness of vascular plants based on Yang *et al.*^42^ and Y-axis is the number of vascular plant species in the dataset of this study minus that of Yang *et al.*^42^. The mean and standard deviation in the difference in the number of vascular plant species between the two datasets were 305.2 and 314.3, respectively. Among the 2010 ‘under-sampled’ counties based on the definition of Yang *et al.*^42^, the inventory completeness in 87% of counties was greatly improved.

**Figure 4:**
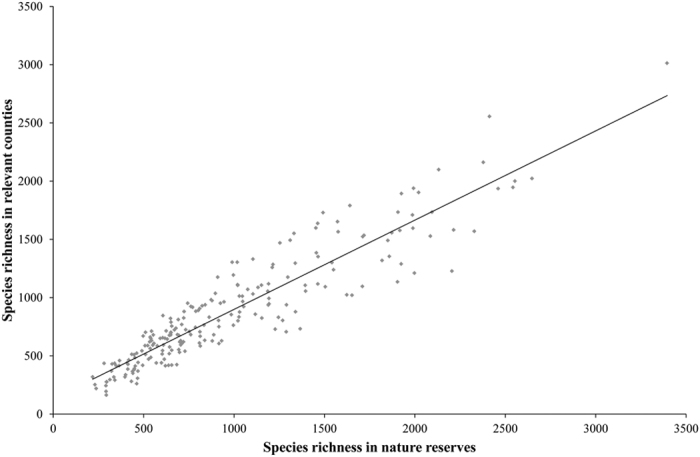
Comparison between the number of vascular plant species in 217 counties in the dataset of this study and the number of vascular plant species in 217 relevant nature reserves. The species numbers of vascular plant species in nature reserves were compiled from the full-surveyed information. These 217 nature reserves are nested within the relevant counties. The number of vascular plant species in relevant counties and nature reserves was basically approximate (r = 0.92, P < 0.01).

**Figure 5:**
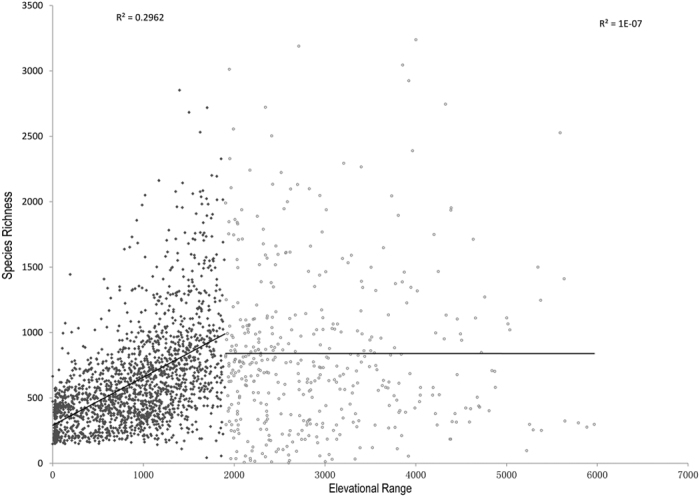
Relationship between elevational range and vascular plant species richness. Counties with elevational range larger than 6,000 m were excluded in the analysis. The relationship is significant when elevational range is less than 1,900 m (R^2^ = 0.2962, F = 815.2, P < 0.001), beyond which the relationship is marginal (R^2^ = 0.0000, F = 0.02, P = 0.89).

**Figure 6:**
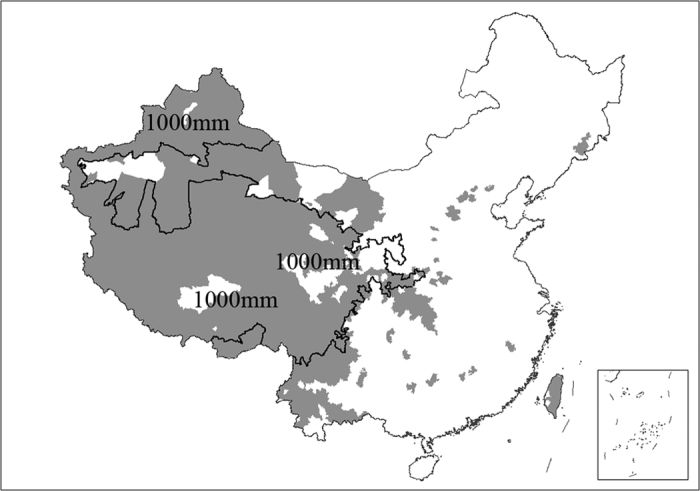
Areas (grey) with elevational range larger than 1,900 m mainly distributed in southwestern and northwestern part of China. Line indicates low-energy areas where annual potential evapotranspiration is around 1,000 mm. The inset in the right bottom of the figure shows the southern boundary of China, including all islands in the South China Sea. Data on national territory were from the National Administration of Surveying, Mapping and Geoinformation of China (http://www.sbsm.gov.cn/). The map was created using the software Arc GIS 9.3 which was purchased from ESRI-China (http://www.esrichina-bj.cn/) by Nanjing Institute of Environmental Sciences affiliated to the Ministry of Environmental Protection of China, with user number C-801668.

We studied the species richness in the terrestrial and inland water ecosystems across China. To get insight into this issue at the regional scale, we further made research in the major three regions (EMR, NAR and QTR) based on Zhao’s geographical regionalization system^31^ (Fig. 1), because the obvious disparity in climatological, geographical and topographical features makes the unique distribution pattern of biodiversity and the different responses of biodiversity to climate change and human intervention occur in the three regions^31^,^34^,^78^,^79^. EMR, accounting for about 46.6% of the total terrestrial area of China, dominated by monsoon climate, is characterized by humid climate, significant change in temperature along latitude and great human intervention, with elevation mostly lower than 1,000 m. NAR, accounting for 29.1% of the total terrestrial area of China, mostly grasslands and deserts, is mainly of arid and semi-arid climate, with high precipitation variability, plateaus of approximately 1,000 m, and some mountains higher than 3,000 m. QTR, accounting for 24.3% of the total terrestrial area of China, harbors the world’s largest and highest plateau, with an average elevation of more than 4,000 m, thin air, low temperature and strong wind^52^.

### Species richness data

We created a database of the geographical distribution of 30,519 vascular plant species and 565 mammal species from 2,376 counties in the terrestrial and inland water ecosystems of China. Species in marine ecosystems, exotic species, and cultivated or bred species in botanical gardens, zoos or farms were excluded. The checklist of vascular plants was derived from Species 2000, Catalogue of Life China edited by the Biodiversity Committee, CAS (Checklist 2011)^80^ (Appendix S1). The checklist of mammal species was based on the complete information compiled by Wang^81^ and Jiang *et al.*^82^ (Appendix S2). The species distribution information in the database was mainly compiled from literatures from 1970 to 2012 (including national floras and faunas, e.g., *Flora Reipulicae Popularis Sinicae*^83^ [Supplementary information], *Flora of China*^84^, *Higher Plants of China*^85^ [Supplementary text], *Fauna Sinica*.*Mammalia*^86^,^87^, regional and provincial monographs on floras and faunas, e.g., *Flora Yunnanica*^88^ [Supplementary text], *Mammals of Beijing*^89^, and numerous studies in biodiversity, e.g., the study of Cheng and Xiao^90^) and collection information of specimens in herbaria of more than 20 institutes and universities. Some recent ground observation information of plants and mammals was also integrated into the database based on records of field surveys by experts from more than 11 institutes of the Chinese Academy of Sciences and over 14 universities. We then invited more than 50 experts specialized in different specific taxa to review the information of species distribution across the whole country. In order to get a finer view of species richness, we used ‘county’ as the basic assessment unit. Such units were respectively treated as one assessment unit, i.e., the urban area of a municipality, the urban area of a capital city in a province and autonomous region, the urban area of a city at prefectural level and a special administrative region (e.g., Hong Kong and Macau) because the presence of species in such regions was mostly recorded in the above units instead of county. In total, 2,376 assessment units (thereafter named as counties) were included for the analysis.

### Environmental data

We selected 19 environmental variables that were considered to mostly explain species richness patterns of vascular plants and mammals from previous studies according to the core hypotheses^24^,^25^,^26^,^39^,^91^, including: (1) mean annual precipitation; (2) precipitation of the wettest quarter; (3) precipitation of the driest quarter; (4) mean annual dryness; (5) mean annual temperature; (6) maximum temperature of the warmest month; (7) minimum temperature of the coldest month; (8) annual potential evapotranspiration; (9) annual actual evapotranspiration; (10) net primary productivity; (11) normalized difference vegetation index; (12) mean diurnal range; (13) temperature seasonality; (14) temperature annual range; (15) precipitation seasonality; (16) elevational range; (17) mean elevation; (18) main land cover type, and (19) number of land cover types. These above environmental variables were classified into five categories (hypotheses) (Table S2), i.e. water-energy dynamics hypothesis (environmental variables 1 to 4), ambient energy (temperature) hypothesis (environmental variables 5 to 8), productivity hypothesis (environmental variables 9 to 11), environmental stability hypothesis (environmental variables 12 to 15), and habitat heterogeneity hypothesis (environmental variables 16 to 19). Although species richness gradients are also attributed to differences in evolutionary history according to the historical hypothesis^92^,^93^, we did not test the historical hypothesis in this study as it was difficult to evaluate the historical effects and incorporate historical factors into regression models^94^.

Data on these environmental variables were from public sources that are often used by peer-reviewed literatures. Data on climate variables were from WorldClim-Global Climate Data (http://www.worldclim.org) with a resolution of 30” × 30”. Data on potential evapotranspiration (PET) were from the website of the Consortium for Spatial Information (CGIAR-CSI) of the Consultative Group on International Agricultural Research (http://www.cgiar-csi.org) (30” × 30” resolution). Data on annual actual evapotranspiration (AET) were from the UNEP website (http://www.grid.unep.ch/data/download/gnv183.zip) (0.5° × 0.5° resolution). Data on the normalized difference vegetation index (NDVI) (250 m × 250 m resolution) and topography (90 m × 90 m resolution) were from SRTM90 of Global Land Cover Facility (http://glcf.umd.edu/data/). Data on net primary productivity (NPP) were from the NASA/EOS Project of the University of Montana (http://www.ntsg.umt.edu/project/mod17#data-product) (30” × 30” resolution). Data on land- cover type were from the website of the European Space Agency (http://bioval.jrc.ec.europa.eu/products /glc2000/glc2000.php) (30” × 30” resolution). Data on administrative boundary of counties were from the National Archives for Surveying and Mapping of China (http://ngcc.sbsm.gov.cn/article/en/or/an/). We obtained values of environmental variables in a county by calculating the average value of the variables among all pixels in the county. Elevational range was calculated as the maximum minus the minimum elevation recorded in a county. Main type of land cover was estimated by the majority of land cover type in a county. Mean annual dryness was calculated as the mean annual precipitation divided by the annual potential evapotranspiration^95^. The mean values of environmental variables between 1950 to 2000 were used to conduct multivariate analysis of species richness data^24^.

Data on major environmental variables in the whole country and three separate regions (EMR, NAR and QTR) were listed in Table 2.

### Assessing inventory completeness

We made an assessment of the inventory completeness of vascular plants in this study before the model test in order to control the sampling bias as much as possible. First, we compared the inventory completeness of our database at the county level with that of the study of Yang *et al.*^42^. Due to lack of enough specimen information, we were not able to directly compute the smoothed species accumulation curves^42^. Instead, we calculated the values of ‘mean’ and ‘standard deviation’ in the difference in the number of vascular plant species between the above two datasets. If the data quality of species was improved in the ‘under-sampled’ counties defined by Yang *et al.*^42^ to a larger extent, we considered that the inventory completeness in such counties was improved as much as possible. In addition, we also compared the number of vascular plant species per county among 217 counties in the dataset of this study with species numbers that were compiled from the well-surveyed information from 217 nature reserves. These 217 nature reserves are distributed within relevant 217 counties, respectively. If the species richness of vascular plants among counties in the dataset of our study was basically approximate to that from the well-surveyed nature reserves, we considered that a good level of inventory completeness was achieved in such counties.

### Statistical analyses

In the first step, we performed Spearman (two-sided) correlation analysis between any two environmental variables in each hypothesis in order to reduce multicollinearity among the variables. Besides, we analyzed potential single predictors of species richness using univariate regression models. Then, we identified strongly intercorrelated variables (Spearman’s coefficient >0.7) and retained the variables that explained more deviance in univariate regression models^24^,^96^,^97^. Thus, we selected predictors for each hypothesis and reduced the correlation among predictors in each category (Table S2).

In the second step, we established generalized linear models (GLM) with the selected predictors from the first step^43^, and calculated the statistical significance at the p < 0.05 for the regression coefficient of each variable. This process was replicated 1000 times. We counted the number of times the regression coefficient of each variable reached statistical significance. The top six variables were selected according to the percentage of a variable reaching statistical significance among 1000 times (Table S3).

In the third step, to avoid inflation of type I errors and invalid parameter estimate owning to spatial autocorrelation, we established spatial linear models (SLM) using the six variables selected in the second step. The simultaneous autoregressive (SAR) models were used to account for spatial autocorrelation. Among the three different SAR model types (spatial error = SAR_err_, lagged = SAR_lag_ and mixed = SAR_mix_), we employed SAR_err_ when dealing with spatially autocorrelated species distribution data^98^. We tested a set of possible lag distances (50, 100, 200, 400, 600, 800 and 1,000 km) for each model and determined the degree of spatial autocorrelation in the residuals of models using Moran’ s I coefficient. SAR_err_ with a lag distance of 100 km accounted best for the spatial structure in the data set according to the minimum value of AIC. As r^2^ values are not directly provided for SAR models, we assessed the maximum model fit based on a pseudo-r^2^ value, that is calculated as the squared Pearson correlation coefficient between predicted and observed (species richness) values^98^. By testing z value for its significance, we examined the contribution of each predictor to the residuals of species richness in the best-fit SLM^43^. Finally, we compared multivariate regressions of six predictors with that of 19 variables, and detected if changes in model fit (Δr^2^) occur to assess the robustness of best-fit SLM^43^.

Species richness, areas and environmental variables were log_10_-transformed in all analyses unless otherwise stated. Statistical analyses were carried out using the free software packages R, version 2.15^99^,^100^ unless otherwise stated. As the large changes in county area (mean: 3908.7 km^2^; standard deviation: 9287.6 km^2^) may influence patterns of species richness^93^, we regressed species richness on county area and obtained residuals of species richness^101^, and data on the residuals of species richness were used in these three steps to avoid effects of area. In addition, we analyzed the correlation between elevational range and species richness (counties where elevational range larger than 6,000 m were excluded from the correlation analysis) using split-line regression techniques^6^.

### Area-effect test

Data on the residuals of species richness were used to establish multivariate models, although this may lead to biased parameter estimates^102^. We also established SLM multivariate models using raw data and treated area as a variable in the model. In this process, influence of area on environmental variables (especially elevational range and the number of landcover types) was considered. We found that no obvious difference existed in multivariate models between the two methods (Table S5). Thus, the results from the residuals of species richness were presented in this study.

### Test the impact of sampling bias on the robustness of multivariate models

The performance of multivariate models may be influenced by geographical sampling bias^42^,^64^ though the limitations in the database were reduced to the lowest extent. To test the impact of sampling bias on the robustness of GLM models, we performed a bootstrap procedure with stratified random sampling^74^,^76^,^77^. To ensure the thorough coverage of environmental conditions in the study region, we adopted the stratification system based on the phytogeographic regions in China^103^ for vascular plants and zoogeographical regions in China^52^ for mammals. Two principles were observed in this procedure: the first is that the target regions (i.e., whole China, EMR, NAR and QTR respectively) should remain unchanged, and the second is that sampling units (i.e., the basic assessment unit) should be randomly selected^41^. We illustrated the procedure as follows: (1) Stratified random sampling was used to generate a sample of 60% of the total dataset from each strata of target regions (whole China, EMR, NAR and QTR respectively)^77^; (2) We fitted a GLM model based on the subset of data (60%); (3) When p < 0.05, the regression coefficient is considered statistically significant; (4) The above steps from (1) to (3) were repeated 1000 times with randomly generated samples. We summed the number of times each variable reached the statistical significance based on the regression coefficient and selected the top six variables based on the percentage of a variable reaching statistical significance in the procedure with 1000 replicates; (5) We then randomly resampled 70%, 80% and 90% of total dataset respectively, and repeated the above steps from (1) to (4). If consistent environmental variables were finally obtained from GLM models based on different proportions of samples (60%, 70%, 80% and 90%), as compared with that of multivariate models in the target regions (100%), we can effectively control the impact of sampling bias on the models using stratified random sampling and verify the robustness of multivariate models.

## Additional Information

**How to cite this article**: Xu, H. *et al.* Disentangling the determinants of species richness of vascular plants and mammals from national to regional scales. *Sci. Rep.* **6**, 21988; doi: 10.1038/srep21988 (2016).

## Supplementary Material

Supplementary Appendix S1

Supplementary Appendix S2

Supplementary Information

## Figures and Tables

**Table 1:** SLM multivariate models for the residuals of species richness of vascular plants and mammals in the terrestrial and inland water ecosystems of China.

Model	Predictors		Whole China	EMR	NAR	QTR
			**Vascular plants**			
Model with 6 predictors	Elevational range	z	15.68***	9.19***	6.34***	4.15***
	Net primary productivity	z	4.83***	—	—	5.59***
	Normalized difference vegetation index	z	—	—	5.47***	—
	Maximum temperature of the warmest month	z	3.64***	−2.72**	—	—
	Minimum temperature of the coldest month	z	—	6.22***	—	−1.72
	Annual potential evapotranspiration	z	—	−1.95	—	—
	Mean diurnal range	z	—	—	0.85	—
	Temperature annual range	z	—	—	—	0.03
	Mean annual precipitation	z	6.80***	—	—	—
	Precipitation of the driest quarter	z	—	—	−0.33	1.37
	Precipitation seasonality	z	−3.83***	−2.35*	−1.79	−2.18*
	Main land cover type	z	−4.11***	−2.23*	−1.48	—
	AIC		−1147.1	−1606.7	−147.5	153.6
	Fitted values	r^2^	0.61	0.58	0.53	0.51
	Moran’s I		−0.06	−0.02	−0.08	0.00
19-predictor model	AIC		−1202.6	−1626.8	−148.7	167.4
	Fitted values	r^2^	0.62	0.60	0.58	0.55
			**Mammals**			
Model with 6 predictors	Elevational range	z	9.59***	6.71***	3.54***	3.79***
	Mean annual precipitation	z	—	6.22***	—	—
	Precipitation of the driest quarter	z	—	—	0.62	—
	Precipitation of the wettest quarter	z	—	—	−1.17	—
	Net primary productivity	z	8.65***	0.10	—	4.46***
	Mean annual dryness	z	—	—	—	2.39*
	Normalized difference vegetation index	z	—	—	3.27**	—
	Maximum temperature of the warmest month	z	2.67***	−4.83***	—	—
	Minimum temperature of the coldest month	z	—	—	—	−0.59
	Temperature annual range	z	−2.64**	—	—	—
	Precipitation seasonality	z	−2.09*	1.12	−0.44	−4.05***
	Main land cover type	z	−7.11***	−4.02***	—	—
	Number of land cover types	z	—	—	−0.21	—
	Mean elevation	z	—	—	—	−2.09*
	AIC		−1505.0	−1443.3	−170.5	−83.4
	Fitted values	r^2^	0.53	0.54	0.36	0.68
	Moran’s I		−0.02	−0.01	−0.00	0.01
19-predictor model	AIC		−1524.2	−1446.4	−173.7	−70.0
	Fitted values	r^2^	0.54	0.55	0.43	0.68

We divided the area of China into three regions: the Eastern Monsoon Region (EMR, n = 1995), the Northwestern Arid Region (NAR, n = 210), and the Qinghai-Tibet Plateau Region (QTR, n = 171). Species richness and all continuous variables were log_10_-transformed. Data on residuals of species richness were used to remove the effects of area (*Pr(>|z|) <0.05, ** Pr(>|z|) <0.01, ***Pr(>|z|) <0.001).

**Table 2:** Summary of main environmental variables in China and its three regions.

Main environmental variables	Whole China		EMR		NAR		QTR	
	Mean	SD	Mean	SD	Mean	SD	Mean	SD
Mean annual precipitation (mm)	921.3	488.0	1029.1	448.9	253.6	136.6	483.4	249.3
Precipitation of the wettest quarter (mm)	474.0	206.5	522.7	180.8	158.8	97.0	293.0	130.8
Precipitation of the driest quarter (mm)	60.7	58.6	70.4	59.1	9.1	7.7	11.0	9.2
Mean annual dryness	85.3	40.1	93.2	37.1	28.8	16.7	63.2	28.0
Mean annual temperature (0.1 °C)	123.5	61.9	140.5	49.6	54.5	32.3	10.0	35.7
Max temperature of the warmest month (0.1 °C)	287.6	45.4	299.5	28.1	274.9	29.8	162.8	30.4
Min temperature of the coldest month (0.1 °C)	−58.9	102.7	−34.9	92.7	−189.8	39.6	−178.5	48.6
Annual potential evapotranspiration (mm)	1055.8	157.4	1091.4	130.7	952.1	134.3	767.1	109.3
Net primary productivity (gC·m^−2^·a^−1^)	437.9	231.8	488.6	208.8	129.1	79.4	225.3	195.7
Annual actual evapotranspiration (mm)	713.1	274.8	780.5	232.3	258.8	137.8	485.9	194.9
Normalized difference vegetation index	494.7	135.0	527.6	98.5	303.8	163.2	346.0	167.3
Temperature annual range (0.1 °C)	346.5	86.4	334.4	83.9	464.7	31.3	341.3	38.2
Mean diurnal range (0.1 °C)	101.5	23.7	95.4	20.0	130.5	10.6	137.6	14.8
Temperature seasonality	8725.3	2635.6	8522.7	2573.9	11971.4	1243.9	7103.1	1154.4
Precipitation seasonality	81.3	23.5	78.7	23.0	90.6	22.6	100.5	17.9
Elevational range (m)	1247.3	1086.0	1024.8	812.1	1884.0	1533.8	3060.8	1211.3
Mean elevation (m)	860.5	1100.9	541.7	609.4	1308.9	515.4	4029.8	842.7
Number of land cover types	6.5	2.1	6.4	2.1	6.8	2.4	7.0	2.2

EMR: Eastern Monsoon Region, n = 1995; NAR: Northwestern Arid Region, n = 210; QTR: Qinghai-Tibet Plateau Region, n = 171. There are significant differences (one-way ANOVA analysis, p < 0.05) in means of these variables among the three regions.
