# Supplementary material for: Disentangling the determinants of species richness of vascular plants and mammals from national to regional scales
Source: Sci Rep. 2016 Feb 23;6:21988. doi: 10.1038/srep21988 (PMC4763236; doi:10.1038/srep21988)
Supplement: Supplementary Information [file srep21988-s3.pdf]

Supplementary Information

Disentangling the determinants of species richness of vascular plants and mammals from national to regional scales

Haigen Xu[1*], Mingchang Cao[1], Yi Wu[2], Lei Cai[3], Yun Cao[1, 4], Jun Wu[1], Juncheng Lei[2], Zhifang Le[1], Hui Ding[1], Peng Cui[1]

(Initials of author names: Xu HG, Cao MC, Wu Y, Cai L, Cao Y, Wu J, Lei JC, Le ZF, Ding H, Cui P)

[1]Nanjing Institute of Environmental Sciences, Ministry of Environmental Protection, Nanjing 210042, China.

[2]College of Forest Resources and Environment, Nanjing Forestry University, Nanjing 210037, China.

[3]Department of Natural Ecology Conservation, Ministry of Environmental Protection, Beijing 100035, China.

[4] Department of Biology, Nanjing University, Nanjing 210093, China.

*Corresponding author: xhg@nies.org and xuhgs@sina.com, Tel:(+86-25)85287081

Contents

Supplementary text (Errors in species richness data and its treatment)

Table S1 Correlations between environmental variables in whole China and its three regions

Table S2 Variables selected based on Spearman (two-sided) correlation analysis and univariate regression for the residuals of species richness of vascular plants and mammals in whole China and its three regions

Table S3 Top six predictors selected based on the percentage of a variable reaching statistical significance among 1000 replicates

Table S4 Top six predictors selected based on stratified random sampling

Table S5 SLM multivariate models for species richness of vascular plants across China and its three regions with area as a variable in the models

Fig. S1 Map of assessment units (counties) in China

Appendix S1 Checklist of vascular plants

Appendix S2 Checklist of mammals

Supplementary text

Errors in species richness data

Although this is one of the best datasets ever developed in China, it has some sampling bias that may introduce errors into the results of our study. Two types of errors are possible:

(1) **Omission errors,** which occur when a given species is not recorded in a county while, in fact, it is present in the county, and;

(2) **Commission errors,** which occur when a given species is recorded in a county when, in fact, it is not present in the county.

We discuss the errors in the dataset to better understand the results and implications of our study. The errors are not exclusive to our dataset– in fact they are prevalent among most published species distribution databases at all scales[1-7]. The current datasets are much more prone to omission errors.

Omission errors

A common shortcoming in the current databases is that sampling efforts are not uniform in space[5,8-10]. Most species records are derived from opportunistic collections without a harmonious sampling strategy to cover the full variation of environmental conditions in the entire target region. Sampling bias tends to be higher for certain taxonomic groups or regions. Some species receive special attention, or are easy to be detected. Some regions are close to researchers, or have more research funding[7,11]. Biases are very common as records can be spatially biased towards more popular species or easily accessible sites[5,12]. There are a lot of species that are actually present but have never yet been recorded. Poorly known species and regions are most likely to be affected by the above limitations. Detailed surveys across the entire possible range of most species are barely conducted due to lack of resources[13-14]. Few counties in China have been surveyed with the aim of generating complete species lists[15]. Consequently, omission errors exist in the data on species distribution in some counties of China due to incomplete documentation of specie distribution[15].
Well-known regions are less likely to have omission errors.

Commission errors

Species may be misidentified, or the locations may be wrongly recorded, which result in commission errors[5,11]. Species occurrence is not fixed especially for mammals as they move dynamically in time and space as a consequence of changing biotic and abiotic conditions. Due to change of habitats or land use, such as transformation of forests into croplands, and wetlands into rice paddies, the distribution of species is likely to change. Thus, data on species distribution based on literatures or specimens may overestimate species locations and then lead to commission errors in this analysis.

Treatment with errors in species richness data

Data sources and peer-review

Data on species distribution were collected from three sources: (i) approximately 600 literatures (the most complete literature we can find as far as possible) about the distribution of vascular plants and mammals from 1970 to 2012, including national floras and faunas, e.g., *Flora Reipulicae Popularis Sinicae*[16-141], *Flora of China*[142], *Higher Plants of China*[143-154], *Fauna Sinica · Mammalia*[155-156], regional and provincial monographs on floras and faunas, e.g., *Flora Yunnanica*[157-177], *Mammals of Beijing*[178], and numerous studies in biodiversity, e.g., the study of Cheng and Xiao[179]; (ii) collection information of specimens in herbaria of more than 20 institutes and universities; and (iii) some recent ground observation information of vascular plants and mammals based on records of field surveys by experts from more than 11 institutes of the Chinese Academy of Sciences and over 14 universities. Among them, 80% of data on species distribution were from the literatures, 15% from collection information of specimens, and 5% from ground observation. To improve the data quality, we organized more than 20 expert meetings and invited over 50 experts specialized in different specific taxa to review the data of spatial distribution of each species across the country based on a GIS information system that we developed for species distribution at the county level. These experts specialized in specific taxa are from more than 11 institutes of the Chinese Academy of Sciences (e. g., Institute of Botany, Kunming Institute of Botany, South China Botanical Garden, Wuhan Botanical Garden, Institute of Applied Ecology, Jiangsu Institute of Botany, Guangxi Institute of Botany, Institute of Zoology, Kunming Institute of Zoology, Chengdu

Institute of Biology, and Northwest Institute of Plateau Biology) and over 14 universities (e.g., Wuhan University, Xiamen University, Sichuan University, Northwest University, Southwest University, Guangzhou University, Northwest Normal University, Chongqing Normal University, Qufu Normal University, Northeast Forestry University, Nanjing Agricultural University, Nanjing Forestry University, South China Agricultural University, and Xinjiang Agricultural University, etc.). Through the above process, we comprehensively collected the data on species geographical distribution at county level and improved the accuracy of species distribution as far as possible.

Using stratified random sampling to test the robustness of multivariate models

Although limitations in the database were reduced to the lowest extent, we performed a bootstrap procedure with stratified random sampling to test the impact of sampling bias on the robustness of the final (GLM) model [180-183].We illustrated the procedures as follows:

(1) We implemented a stratified random sampling procedure to generate a sample of 60% of the total dataset from target regions (whole China, EMR, NAR and QTR respectively). The stratification system was based on the phytogeographic regions in China[184] for vascular plants and zoogeographical regions in China[185] for mammals.

(2) We fitted a GLM model based on the subset data (60%).

(3) We assigned the statistical significance at $p<0.05$ according to the regression coefficient of each variable.

(4) The above steps from (1) to (3) were replicated 1000 times with randomly generated samples. We summed the number of times each variable reached the statistical significance based on the regression coefficient. The top six variables were selected according to the number of times of a variable that reached statistical significance among 1000 times.

(5) We then randomly resampled 70%, 80% and 90% of total dataset respectively, and repeated the steps from (1) to (4).

(6) If consistent environmental variables were finally obtained from the above models based on different proportions of samples (60%, 70%, 80% and 90%), as compared with that of multivariate models in target regions (100%), we can effectively control the impact of sampling bias and verify the robustness of multivariate models.

Through the above analysis, we found that consistent results were obtained in GLM models based on different proportions of samples (from 60% to 90%), as compared with that of multivariate models in target regions (100%) (Table S4). For example ,in all GLM models for plants in whole China, the top six environmental variables that reached statistical significance for the most times remained unchanged from 60% to 100%, i.e., elevational range, net primary productivity, precipitation seasonality, mean annual precipitation, maximum temperature of the warmest month, and main land cover type (Table S4). It suggests that the impact of sampling bias on multivariate models was effectively controlled and the multivariate models were robust.

References

1. Balmford, A., Moore, J. L., Brooks, T., Burgess, N., Hansen, L. A., Williams, P. & Rahbek, C. Conservation conflicts across Africa. Science 291: 2616-2619 (2001).
2. Burgess, N. D., Rahbek, C., Larsen, F. W., Williams, P. & Balmford, A. How much of the vertebrate diversity of sub-Saharan Africa is catered for by recent conservation proposals? Biol. Conserv. 107: 327-339 (2002).
3. Andelman, S. J. & Willig, M. R. Present patterns and future prospects for biodiversity in the Western Hemisphere. Eco. Lett. 6: 818-824 (2003).
4. Rodrigues, A. S., Andelman, S. J., Bakarr, M. I., Boitani, L. et al. Effectiveness of the global protected area network in representing species diversity. Nature 428:640-643 (2004).
5. Boitani, L., Maiorano, L., Baisero, D, Falcucci, A., Visconti, P., and Rondinini, C. What spatial data do we need to develop global mammal conservation strategies? Phil. Trans. R. Soc. B 366: 2623-2632 (2011).
6. Chen, G., Kéry, M., Plattner, M., Ma, K. and Gardner, B. Imperfect detection is the rule rather than the exception in plant distribution studies. Journal of Ecology 101: 183-191 (2013).
7. Meyer, C., Kreft, H., Guralnick, R. & Jetz, W. Global priorities for an effective information basis of biodiversity distributions. Nature Communications 6:8221 (2015).
8. Hortal, J., Lobo, J.M. & Jim_enez-Valverde, A. Limitations of biodiversity

databases: case study on seed-plant diversity in Tenerife, Canary Islands. Conservation Biology 21: 853-863 (2007).

9. Soberón, J., Jim_enez, R., Golubov, J. & Koleff, P. Assessing completeness of biodiversity databases at different spatial scales. Ecography 30: 152-160 (2007).
10. Beck, J. and Schwanghart, W. Comparing measures of species diversity from incomplete inventories: an update. Methods in Ecology and Evolution 1: 38-44 (2010).
11. Rondinini, C., Wilson, K. A., Boitani, L., Grantham, H. and Possingham, H.P. Tradeoffs of different types of species occurrence data for use in systematic conservation planning. Ecology Letters 9: 1136-1145 (2006).
12. Boakes, E. H., McGowan, P. J. K., Fuller, R. A., Changqing, D., Clark, N. E., O'Connor, K. & Mace, G. M. Distorted views of biodiversity: spatial and temporal bias in species occurrence data. PLoS Biol. 8: e1000385 (2010).
13. Haila, Y. & Margules, C.R. Survey research in conservation biology. Ecography 19: 323-331 (1996).
14. Pressey, R.L. Conservation planning and biodiversity: assembling the best data for the job. Conserv. Biol. 18: 1677-1681 (2004).
15. Qian, H. and Ricklefs, R. E. Latitude, tree species diversity and the metabolic theory of ecology. Global Ecology and Biogeography 20: 362-365 (2011)
16. Editorial Committee of Flora Reipublicae Popularis Sinicae. Flora Reipulicae Popularis Sinicae (Vol. 1). Science Press, Beijing，pp.1-1044(2004).
17. Editorial Committee of Flora Reipublicae Popularis Sinicae. Flora Reipulicae Popularis Sinicae (Vol. 2). Science Press, Beijing，pp.1-379(1959).
18. Editorial Committee of Flora Reipublicae Popularis Sinicae. Flora Reipulicae Popularis Sinicae (Vol. 3(1)). Science Press, Beijing，pp.1-279(1990).
19. Editorial Committee of Flora Reipublicae Popularis Sinicae. Flora Reipulicae Popularis Sinicae (Vol. 3(2)). Science Press, Beijing，pp.1-511(1999).
20. Editorial Committee of Flora Reipublicae Popularis Sinicae. Flora Reipulicae Popularis Sinicae (Vol. 4(1)). Science Press, Beijing，pp.1-353(1999).
21. Editorial Committee of Flora Reipublicae Popularis Sinicae. Flora Reipulicae Popularis Sinicae (Vol. 4(2)). Science Press, Beijing，pp.1-238(1999).
22. Editorial Committee of Flora Reipublicae Popularis Sinicae. Flora Reipulicae Popularis Sinicae (Vol. 5(1)). Science Press, Beijing，pp.1-220(2000).

23. Editorial Committee of Flora Reipublicae Popularis Sinicae. Flora Reipulicae Popularis Sinicae (Vol. 5(2)). Science Press, Beijing，pp.1-228(2001).
24. Editorial Committee of Flora Reipublicae Popularis Sinicae. Flora Reipulicae Popularis Sinicae (Vol. 6(1)). Science Press, Beijing，pp.1-199(1999).
25. Editorial Committee of Flora Reipublicae Popularis Sinicae. Flora Reipulicae Popularis Sinicae (Vol. 6(2)). Science Press, Beijing，pp.1-349(2000).
26. Editorial Committee of Flora Reipublicae Popularis Sinicae. Flora Reipulicae Popularis Sinicae (Vol. 6(3)). Science Press, Beijing，pp.1-274(2004).
27. Editorial Committee of Flora Reipublicae Popularis Sinicae. Flora Reipulicae Popularis Sinicae (Vol. 7). Science Press, Beijing，pp.1-504(1978).
28. Editorial Committee of Flora Reipublicae Popularis Sinicae. Flora Reipulicae Popularis Sinicae (Vol. 8). Science Press, Beijing，pp.1-193(1992).
29. Editorial Committee of Flora Reipublicae Popularis Sinicae. Flora Reipulicae Popularis Sinicae (Vol. 9(1)). Science Press, Beijing，pp.1-704(1996).
30. Editorial Committee of Flora Reipublicae Popularis Sinicae. Flora Reipulicae Popularis Sinicae (Vol. 9(2)). Science Press, Beijing，pp.1-405(2002).
31. Editorial Committee of Flora Reipublicae Popularis Sinicae. Flora Reipulicae Popularis Sinicae (Vol. 9(3)). Science Press, Beijing，pp.1-329(1987).
32. Editorial Committee of Flora Reipublicae Popularis Sinicae. Flora Reipulicae Popularis Sinicae (Vol. 10(1)). Science Press, Beijing，pp.1-391(1990).
33. Editorial Committee of Flora Reipublicae Popularis Sinicae. Flora Reipulicae Popularis Sinicae (Vol. 10(2)). Science Press, Beijing，pp.1-294(1997).
34. Editorial Committee of Flora Reipublicae Popularis Sinicae. Flora Reipulicae Popularis Sinicae (Vol. 11). Science Press, Beijing，pp.1-235(1961).
35. Editorial Committee of Flora Reipublicae Popularis Sinicae. Flora Reipulicae Popularis Sinicae (Vol. 12). Science Press, Beijing，pp.1-528(2000).
36. Editorial Committee of Flora Reipublicae Popularis Sinicae. Flora Reipulicae Popularis Sinicae (Vol. 13(1)). Science Press, Beijing，pp.1-130(1991).
37. Editorial Committee of Flora Reipublicae Popularis Sinicae. Flora Reipulicae Popularis Sinicae (Vol. 13(2)). Science Press, Beijing，pp.1-211(1979).
38. Editorial Committee of Flora Reipublicae Popularis Sinicae. Flora Reipulicae Popularis Sinicae (Vol. 13(3)). Science Press, Beijing，pp.1-260(1997).

39. Editorial Committee of Flora Reipublicae Popularis Sinicae. Flora Reipulicae Popularis Sinicae (Vol. 14). Science Press, Beijing，pp.1-286(1980).
40. Editorial Committee of Flora Reipublicae Popularis Sinicae. Flora Reipulicae Popularis Sinicae (Vol. 15). Science Press, Beijing，pp.1-255(1978).
41. Editorial Committee of Flora Reipublicae Popularis Sinicae. Flora Reipulicae Popularis Sinicae (Vol. 16(1)). Science Press, Beijing，pp.1-198(1985).
42. Editorial Committee of Flora Reipublicae Popularis Sinicae. Flora Reipulicae Popularis Sinicae (Vol. 16(2)). Science Press, Beijing，pp.1-176(1981).
43. Editorial Committee of Flora Reipublicae Popularis Sinicae. Flora Reipulicae Popularis Sinicae (Vol. 17). Science Press, Beijing，pp.1-499(2005).
44. Editorial Committee of Flora Reipublicae Popularis Sinicae. Flora Reipulicae Popularis Sinicae (Vol. 18). Science Press, Beijing，pp.1-412(1999).
45. Editorial Committee of Flora Reipublicae Popularis Sinicae. Flora Reipulicae Popularis Sinicae (Vol. 19). Science Press, Beijing，pp.1-175(1999).
46. Editorial Committee of Flora Reipublicae Popularis Sinicae. Flora Reipulicae Popularis Sinicae (Vol. 20(1)). Science Press, Beijing，pp.1-95(1982).
47. Editorial Committee of Flora Reipublicae Popularis Sinicae. Flora Reipulicae Popularis Sinicae (Vol. 20(2)). Science Press, Beijing，pp.1-381(1984).
48. Editorial Committee of Flora Reipublicae Popularis Sinicae. Flora Reipulicae Popularis Sinicae (Vol. 21). Science Press, Beijing，pp.1-137(1979).
49. Editorial Committee of Flora Reipublicae Popularis Sinicae. Flora Reipulicae Popularis Sinicae (Vol. 22). Science Press, Beijing，pp.1-416(1998).
50. Editorial Committee of Flora Reipublicae Popularis Sinicae. Flora Reipulicae Popularis Sinicae (Vol. 23(1)). Science Press, Beijing，pp.1-224(1998).
51. Editorial Committee of Flora Reipublicae Popularis Sinicae. Flora Reipulicae Popularis Sinicae (Vol. 23(2)). Science Press, Beijing，pp.1-404(1995).
52. Editorial Committee of Flora Reipublicae Popularis Sinicae. Flora Reipulicae Popularis Sinicae (Vol. 24). Science Press, Beijing，pp.1-268(1988).
53. Editorial Committee of Flora Reipublicae Popularis Sinicae. Flora Reipulicae Popularis Sinicae (Vol. 25(1)). Science Press, Beijing，pp.1-209(1998).
54. Editorial Committee of Flora Reipublicae Popularis Sinicae. Flora Reipulicae Popularis Sinicae (Vol. 25(2)). Science Press, Beijing，pp.1-241(1979).

55. Editorial Committee of Flora Reipublicae Popularis Sinicae. Flora Reipulicae Popularis Sinicae (Vol. 26). Science Press, Beijing，pp.1-448(1996).
56. Editorial Committee of Flora Reipublicae Popularis Sinicae. Flora Reipulicae Popularis Sinicae (Vol. 27). Science Press, Beijing，pp.1-621(1979).
57. Editorial Committee of Flora Reipublicae Popularis Sinicae. Flora Reipulicae Popularis Sinicae (Vol. 28). Science Press, Beijing，pp.1-390(1980).
58. Editorial Committee of Flora Reipublicae Popularis Sinicae. Flora Reipulicae Popularis Sinicae (Vol. 29). Science Press, Beijing，pp.1-310(2001).
59. Editorial Committee of Flora Reipublicae Popularis Sinicae. Flora Reipulicae Popularis Sinicae (Vol. 30(1)). Science Press, Beijing，pp.1-273(1996).
60. Editorial Committee of Flora Reipublicae Popularis Sinicae. Flora Reipulicae Popularis Sinicae (Vol. 30(2)). Science Press, Beijing，pp.1-204(1979).
61. Editorial Committee of Flora Reipublicae Popularis Sinicae. Flora Reipulicae Popularis Sinicae (Vol. 31). Science Press, Beijing，pp.1-479(1982).
62. Editorial Committee of Flora Reipublicae Popularis Sinicae. Flora Reipulicae Popularis Sinicae (Vol. 32). Science Press, Beijing，pp.1-545(1999).
63. Editorial Committee of Flora Reipublicae Popularis Sinicae. Flora Reipulicae Popularis Sinicae (Vol. 33). Science Press, Beijing，pp.1-453(1987).
64. Editorial Committee of Flora Reipublicae Popularis Sinicae. Flora Reipulicae Popularis Sinicae (Vol. 34(1)). Science Press, Beijing，pp.1-220(1984).
65. Editorial Committee of Flora Reipublicae Popularis Sinicae. Flora Reipulicae Popularis Sinicae (Vol. 34(2)). Science Press, Beijing，pp.1-279(1992).
66. Editorial Committee of Flora Reipublicae Popularis Sinicae. Flora Reipulicae Popularis Sinicae (Vol. 35(1)). Science Press, Beijing，pp.1-362(1995).
67. Editorial Committee of Flora Reipublicae Popularis Sinicae. Flora Reipulicae Popularis Sinicae (Vol. 35(2)). Science Press, Beijing，pp.1-121(1979).
68. Editorial Committee of Flora Reipublicae Popularis Sinicae. Flora Reipulicae Popularis Sinicae (Vol. 36). Science Press, Beijing，pp.1-405(1974).
69. Editorial Committee of Flora Reipublicae Popularis Sinicae. Flora Reipulicae Popularis Sinicae (Vol. 37). Science Press, Beijing，pp.1-476(1985).
70. Editorial Committee of Flora Reipublicae Popularis Sinicae. Flora Reipulicae Popularis Sinicae (Vol. 38). Science Press, Beijing，pp.1-150(1986).

71. Editorial Committee of Flora Reipublicae Popularis Sinicae. Flora Reipulicae Popularis Sinicae (Vol. 39). Science Press, Beijing，pp.1-218(1988).
72. Editorial Committee of Flora Reipublicae Popularis Sinicae. Flora Reipulicae Popularis Sinicae (Vol. 40). Science Press, Beijing，pp.1-326(1994).
73. Editorial Committee of Flora Reipublicae Popularis Sinicae. Flora Reipulicae Popularis Sinicae (Vol. 41). Science Press, Beijing，pp.1-362(1995).
74. Editorial Committee of Flora Reipublicae Popularis Sinicae. Flora Reipulicae Popularis Sinicae (Vol. 42(1)). Science Press, Beijing，pp.1-349(1993).
75. Editorial Committee of Flora Reipublicae Popularis Sinicae. Flora Reipulicae Popularis Sinicae (Vol. 42(2)). Science Press, Beijing，pp.1-424(1998).
76. Editorial Committee of Flora Reipublicae Popularis Sinicae. Flora Reipulicae Popularis Sinicae (Vol. 43(1)). Science Press, Beijing，pp.1-145(1998).
77. Editorial Committee of Flora Reipublicae Popularis Sinicae. Flora Reipulicae Popularis Sinicae (Vol. 43(2)). Science Press, Beijing，pp.1-213(1997).
78. Editorial Committee of Flora Reipublicae Popularis Sinicae. Flora Reipulicae Popularis Sinicae (Vol. 43(3)). Science Press, Beijing，pp.1-207(1997).
79. Editorial Committee of Flora Reipublicae Popularis Sinicae. Flora Reipulicae Popularis Sinicae (Vol. 44(1)). Science Press, Beijing，pp.1-188(1994).
80. Editorial Committee of Flora Reipublicae Popularis Sinicae. Flora Reipulicae Popularis Sinicae (Vol. 44(2)). Science Press, Beijing，pp.1-185(1996).
81. Editorial Committee of Flora Reipublicae Popularis Sinicae. Flora Reipulicae Popularis Sinicae (Vol. 44(3)). Science Press, Beijing，pp.1-128(1997).
82. Editorial Committee of Flora Reipublicae Popularis Sinicae. Flora Reipulicae Popularis Sinicae (Vol. 45(1)). Science Press, Beijing，pp.1-140(1980).
83. Editorial Committee of Flora Reipublicae Popularis Sinicae. Flora Reipulicae Popularis Sinicae (Vol. 45(2)). Science Press, Beijing，pp.1-266(1999).
84. Editorial Committee of Flora Reipublicae Popularis Sinicae. Flora Reipulicae Popularis Sinicae (Vol. 45(3)). Science Press, Beijing，pp.1-188(1999).
85. Editorial Committee of Flora Reipublicae Popularis Sinicae. Flora Reipulicae Popularis Sinicae (Vol. 46). Science Press, Beijing，pp.1-291(1981).
86. Editorial Committee of Flora Reipublicae Popularis Sinicae. Flora Reipulicae Popularis Sinicae (Vol. 47(1)). Science Press, Beijing，pp.1-132(1985).

87. Editorial Committee of Flora Reipublicae Popularis Sinicae. Flora Reipulicae Popularis Sinicae (Vol. 47(2)). Science Press, Beijing，pp.1-220(2001).
88. Editorial Committee of Flora Reipublicae Popularis Sinicae. Flora Reipulicae Popularis Sinicae (Vol. 48(1)). Science Press, Beijing，pp.1-157(1982).
89. Editorial Committee of Flora Reipublicae Popularis Sinicae. Flora Reipulicae Popularis Sinicae (Vol. 48(2)). Science Press, Beijing，pp.1-268(1998).
90. Editorial Committee of Flora Reipublicae Popularis Sinicae. Flora Reipulicae Popularis Sinicae (Vol. 49(1)). Science Press, Beijing，pp.1-123(1989).
91. Editorial Committee of Flora Reipublicae Popularis Sinicae. Flora Reipulicae Popularis Sinicae (Vol. 49(2)). Science Press, Beijing，pp.1-334(1984).
92. Editorial Committee of Flora Reipublicae Popularis Sinicae. Flora Reipulicae Popularis Sinicae (Vol. 49(3)). Science Press, Beijing，pp.1-251(1998).
93. Editorial Committee of Flora Reipublicae Popularis Sinicae. Flora Reipulicae Popularis Sinicae (Vol. 50(1)). Science Press, Beijing，pp.1-194(1998).
94. Editorial Committee of Flora Reipublicae Popularis Sinicae. Flora Reipulicae Popularis Sinicae (Vol. 50(2)). Science Press, Beijing，pp.1-179(1983).
95. Editorial Committee of Flora Reipublicae Popularis Sinicae. Flora Reipulicae Popularis Sinicae (Vol. 51). Science Press, Beijing，pp.1-129(1991).
96. Editorial Committee of Flora Reipublicae Popularis Sinicae. Flora Reipulicae Popularis Sinicae (Vol. 52(1)). Science Press, Beijing，pp.1-402(1999).
97. Editorial Committee of Flora Reipublicae Popularis Sinicae. Flora Reipulicae Popularis Sinicae (Vol. 52(2)). Science Press, Beijing，pp.1-179(1983).
98. Editorial Committee of Flora Reipublicae Popularis Sinicae. Flora Reipulicae Popularis Sinicae (Vol. 53(1)). Science Press, Beijing，pp.1-293(1984).
99. Editorial Committee of Flora Reipublicae Popularis Sinicae. Flora Reipulicae Popularis Sinicae (Vol. 53(2)). Science Press, Beijing，pp.1-154(2000).
100. Editorial Committee of Flora Reipublicae Popularis Sinicae. Flora Reipulicae Popularis Sinicae (Vol. 54). Science Press, Beijing，pp.1-190(1978).
101. Editorial Committee of Flora Reipublicae Popularis Sinicae. Flora Reipulicae Popularis Sinicae (Vol. 55(1)). Science Press, Beijing，pp.1-300(1979).
102. Editorial Committee of Flora Reipublicae Popularis Sinicae. Flora Reipulicae Popularis Sinicae (Vol. 55(2)). Science Press, Beijing，pp.1-268(1985).

103.Editorial Committee of Flora Reipublicae Popularis Sinicae. Flora Reipulicae Popularis Sinicae (Vol. 55(3)). Science Press, Beijing，pp.1-255(1992).
104.Editorial Committee of Flora Reipublicae Popularis Sinicae. Flora Reipulicae Popularis Sinicae (Vol. 56). Science Press, Beijing，pp.1-216(1990).
105.Editorial Committee of Flora Reipublicae Popularis Sinicae. Flora Reipulicae Popularis Sinicae (Vol. 57(1)). Science Press, Beijing，pp.1-212(1999).
106.Editorial Committee of Flora Reipublicae Popularis Sinicae. Flora Reipulicae Popularis Sinicae (Vol. 57(2)). Science Press, Beijing，pp.1-438(1994).
107.Editorial Committee of Flora Reipublicae Popularis Sinicae. Flora Reipulicae Popularis Sinicae (Vol. 57(3)). Science Press, Beijing，pp.1-207(1991).
108.Editorial Committee of Flora Reipublicae Popularis Sinicae. Flora Reipulicae Popularis Sinicae (Vol. 58). Science Press, Beijing，pp.1-133(1979).
109.Editorial Committee of Flora Reipublicae Popularis Sinicae. Flora Reipulicae Popularis Sinicae (Vol. 59(1)). Science Press, Beijing，pp.1-201(1989).
110.Editorial Committee of Flora Reipublicae Popularis Sinicae. Flora Reipulicae Popularis Sinicae (Vol. 59(2)). Science Press, Beijing，pp.1-295(1990).
111.Editorial Committee of Flora Reipublicae Popularis Sinicae. Flora Reipulicae Popularis Sinicae (Vol. 60(1)). Science Press, Beijing，pp.1-154(1987).
112.Editorial Committee of Flora Reipublicae Popularis Sinicae. Flora Reipulicae Popularis Sinicae (Vol. 60(2)). Science Press, Beijing，pp.1-150(1987).
113.Editorial Committee of Flora Reipublicae Popularis Sinicae. Flora Reipulicae Popularis Sinicae (Vol. 61). Science Press, Beijing，pp.1-309(1992).
114.Editorial Committee of Flora Reipublicae Popularis Sinicae. Flora Reipulicae Popularis Sinicae (Vol. 62). Science Press, Beijing，pp.1-418(1988).
115.Editorial Committee of Flora Reipublicae Popularis Sinicae. Flora Reipulicae Popularis Sinicae (Vol. 63). Science Press, Beijing，pp.1-575(1977).
116.Editorial Committee of Flora Reipublicae Popularis Sinicae. Flora Reipulicae Popularis Sinicae (Vol. 64(1)). Science Press, Beijing，pp.1-164(1979).
117.Editorial Committee of Flora Reipublicae Popularis Sinicae. Flora Reipulicae Popularis Sinicae (Vol. 64(2)). Science Press, Beijing，pp.1-236(1989).
118.Editorial Committee of Flora Reipublicae Popularis Sinicae. Flora Reipulicae Popularis Sinicae (Vol. 65(1)). Science Press, Beijing，pp.1-214(1982).

119. Editorial Committee of Flora Reipublicae Popularis Sinicae. Flora Reipulicae Popularis Sinicae (Vol. 65(2)). Science Press, Beijing，pp.1-602(1977).
120. Editorial Committee of Flora Reipublicae Popularis Sinicae. Flora Reipulicae Popularis Sinicae (Vol. 66). Science Press, Beijing，pp.1-592(1977).
121. Editorial Committee of Flora Reipublicae Popularis Sinicae. Flora Reipulicae Popularis Sinicae (Vol. 67(1)). Science Press, Beijing，pp.1-160(1978).
122. Editorial Committee of Flora Reipublicae Popularis Sinicae. Flora Reipulicae Popularis Sinicae (Vol. 67(2)). Science Press, Beijing，pp.1-406(1979).
123. Editorial Committee of Flora Reipublicae Popularis Sinicae. Flora Reipulicae Popularis Sinicae (Vol. 68). Science Press, Beijing，pp.1-420(1963).
124. Editorial Committee of Flora Reipublicae Popularis Sinicae. Flora Reipulicae Popularis Sinicae (Vol. 69). Science Press, Beijing，pp.1-605(1990).
125. Editorial Committee of Flora Reipublicae Popularis Sinicae. Flora Reipulicae Popularis Sinicae (Vol. 70). Science Press, Beijing，pp.1-350(2002).
126. Editorial Committee of Flora Reipublicae Popularis Sinicae. Flora Reipulicae Popularis Sinicae (Vol. 71(1)). Science Press, Beijing，pp.1-390(1999).
127. Editorial Committee of Flora Reipublicae Popularis Sinicae. Flora Reipulicae Popularis Sinicae (Vol. 71(2)). Science Press, Beijing，pp.1-338(1999).
128. Editorial Committee of Flora Reipublicae Popularis Sinicae. Flora Reipulicae Popularis Sinicae (Vol. 72). Science Press, Beijing，pp.1-259(1988).
129. Editorial Committee of Flora Reipublicae Popularis Sinicae. Flora Reipulicae Popularis Sinicae (Vol. 73(1)). Science Press, Beijing，pp.1-280(1986).
130. Editorial Committee of Flora Reipublicae Popularis Sinicae. Flora Reipulicae Popularis Sinicae (Vol. 73(2)). Science Press, Beijing，pp.1-189(1983).
131. Editorial Committee of Flora Reipublicae Popularis Sinicae. Flora Reipulicae Popularis Sinicae (Vol. 74). Science Press, Beijing，pp.1-361(1985).
132. Editorial Committee of Flora Reipublicae Popularis Sinicae. Flora Reipulicae Popularis Sinicae (Vol. 75). Science Press, Beijing，pp.1-391(1979).
133. Editorial Committee of Flora Reipublicae Popularis Sinicae. Flora Reipulicae Popularis Sinicae (Vol. 76(1)). Science Press, Beijing，pp.1-136(1983).
134. Editorial Committee of Flora Reipublicae Popularis Sinicae. Flora Reipulicae Popularis Sinicae (Vol. 76(2)). Science Press, Beijing，pp.1-289(1991).

135.Editorial Committee of Flora Reipublicae Popularis Sinicae. Flora Reipulicae Popularis Sinicae (Vol. 77(1)). Science Press, Beijing，pp.1-329(1999).
136.Editorial Committee of Flora Reipublicae Popularis Sinicae. Flora Reipulicae Popularis Sinicae (Vol. 77(2)). Science Press, Beijing，pp.1-171(1989).
137.Editorial Committee of Flora Reipublicae Popularis Sinicae. Flora Reipulicae Popularis Sinicae (Vol. 78(1)). Science Press, Beijing，pp.1-209(1987).
138.Editorial Committee of Flora Reipublicae Popularis Sinicae. Flora Reipulicae Popularis Sinicae (Vol. 78(2)). Science Press, Beijing，pp.1-213(1999).
139.Editorial Committee of Flora Reipublicae Popularis Sinicae. Flora Reipulicae Popularis Sinicae (Vol. 79). Science Press, Beijing，pp.1-96(1996).
140.Editorial Committee of Flora Reipublicae Popularis Sinicae. Flora Reipulicae Popularis Sinicae (Vol. 80(1)). Science Press, Beijing，pp.1-302(1997).
141.Editorial Committee of Flora Reipublicae Popularis Sinicae. Flora Reipulicae Popularis Sinicae (Vol. 80(2)). Science Press, Beijing，pp.1-75(1997).
142.Wu, Z. Y., Raven, P. H., Hong, D. Y. Flora of China. Missouri Botanical Garden Press, St. Louis. http://flora.huh.harvard.edu/china/mss/treatments.htm (1994-2006) (Date of access: from 21/05/2009 to 3/11/2012).
143.Fu, L. G. et al. Higher Plants of China (Vol. 2). Qingdao Publishing House, Qingdao, pp. 1-825 (2008).
144.Fu, L. G. et al. Higher Plants of China (Vol. 3). Qingdao Publishing House, Qingdao, pp. 1-757 (2000).
145.Fu, L. G. et al. Higher Plants of China (Vol. 4). Qingdao Publishing House, Qingdao, pp. 1-745 (2000).
146.Fu, L. G. et al. Higher Plants of China (Vol. 5). Qingdao Publishing House, Qingdao, pp. 1-775 (2003).
147.Fu, L. G. et al. Higher Plants of China (Vol. 6). Qingdao Publishing House, Qingdao, pp. 1-833 (2003).
148.Fu, L. G. et al. Higher Plants of China (Vol. 7). Qingdao Publishing House, Qingdao, pp. 1-929 (2001).
149.Fu, L. G. et al. Higher Plants of China (Vol. 8). Qingdao Publishing House, Qingdao, pp. 1-790 (2001).
150.Fu, L. G. et al. Higher Plants of China (Vol. 9). Qingdao Publishing House, Qingdao, pp. 1-627 (1999).

151.Fu, L. G. et al. Higher Plants of China (Vol. 10). Qingdao Publishing House, Qingdao, pp. 1-719 (2004).
152.Fu, L. G. et al. Higher Plants of China (Vol. 11). Qingdao Publishing House, Qingdao, pp. 1-826 (2005).
153.Fu, L. G. et al. Higher Plants of China (Vol. 12). Qingdao Publishing House, Qingdao, pp. 1-1227 (2009).
154.Fu, L. G. et al. Higher Plants of China (Vol. 13). Qingdao Publishing House, Qingdao, pp. 1-806 (2002).
155.Gao, Y. T. Fauna Sinica · Mammalia (Vol VIII), Carnivora. Science Press, Beijing, pp. 1-377 (1987).
156.Luo, Z. X. Fauna Sinica · Mammalia (Vol VI), Rodentir, Cricetidae. Science Press, Beijing, pp. 1-522 (2000).
157.Editorial Committee of Flora Yunnanica. Flora Yunnanica (Vol. 1). Science Press, Beijing, pp. 1-870 (1977).
158.Editorial Committee of Flora Yunnanica. Flora Yunnanica (Vol. 2). Science Press, Beijing, pp. 1-889 (1979).
159.Editorial Committee of Flora Yunnanica. Flora Yunnanica (Vol. 3). Science Press, Beijing, pp. 1-795 (1983).
160.Editorial Committee of Flora Yunnanica. Flora Yunnanica (Vol. 4). Science Press, Beijing, pp. 1-823 (1986).
161.Editorial Committee of Flora Yunnanica. Flora Yunnanica (Vol. 5). Science Press, Beijing, pp. 1-809 (1991).
162.Editorial Committee of Flora Yunnanica. Flora Yunnanica (Vol. 6). Science Press, Beijing, pp. 1-910 (1995).
163.Editorial Committee of Flora Yunnanica. Flora Yunnanica (Vol. 7). Science Press, Beijing, pp. 1-888 (1997).
164.Editorial Committee of Flora Yunnanica. Flora Yunnanica (Vol. 8). Science Press, Beijing, pp. 1-778 (1997).
165.Editorial Committee of Flora Yunnanica. Flora Yunnanica (Vol. 9). Science Press, Beijing, pp. 1-807 (2003).
166.Editorial Committee of Flora Yunnanica. Flora Yunnanica (Vol. 10). Science Press, Beijing, pp. 1-929 (2006).
167.Editorial Committee of Flora Yunnanica. Flora Yunnanica (Vol. 11). Science

Press, Beijing, pp. 1-754 (2000).
168. Editorial Committee of Flora Yunnanica. Flora Yunnanica (Vol. 12). Science Press, Beijing, pp. 1-884 (2006).
169. Editorial Committee of Flora Yunnanica. Flora Yunnanica (Vol. 13). Science Press, Beijing, pp. 1-918 (2004).
170. Editorial Committee of Flora Yunnanica. Flora Yunnanica (Vol. 14). Science Press, Beijing, pp. 1-885 (2003).
171. Editorial Committee of Flora Yunnanica. Flora Yunnanica (Vol. 15). Science Press, Beijing, pp. 1-874 (2003).
172. Editorial Committee of Flora Yunnanica. Flora Yunnanica (Vol. 16). Science Press, Beijing, pp. 1-876 (2005).
173. Editorial Committee of Flora Yunnanica. Flora Yunnanica (Vol. 17). Science Press, Beijing, pp. 1-650 (2000).
174. Editorial Committee of Flora Yunnanica. Flora Yunnanica (Vol. 18). Science Press, Beijing, pp. 1-525 (2002).
175. Editorial Committee of Flora Yunnanica. Flora Yunnanica (Vol. 19). Science Press, Beijing, pp. 1-681 (2005).
176. Editorial Committee of Flora Yunnanica. Flora Yunnanica (Vol. 20). Science Press, Beijing, pp. 1-785 (2006).
177. Editorial Committee of Flora Yunnanica. Flora Yunnanica (Vol. 21). Science Press, Beijing, pp. 1-477 (2004).
178. Chen, W., Gao, W., Fu, B. Q. Mammals of Beijing. Beijing Press, Beijing, pp. 1-304 (2002).
179. Cheng, R. M., Xiao, W. F. Biodiversity of main coniferous forests at low elevation of Three Gorges Reservoir Area. Chinese Journal of Applied Ecology 16: 1791-1794 (2005).
180. Austin, P. C. and Tu, J. V. Bootstrap methods for developing predictive models. Am. Stat. 58: 131-137 (2004).
181. Rizopoulos, D. bootStepAIC: Model selection by bootstrapping the stepAIC() procedure R package version 1.2-0. 2009. Available: http://cran.r-project.org. Accessed on 2015-02-19.
182. Martins, I.S., Proença, V., Pereira, H.M. The unusual suspect: Land use is a key predictor of biodiversity patterns in the Iberian Peninsula. Acta Oecologica 61:41-50 (2014).

183. Tille, Y. Sampling: Functions for drawing and calibrating samples. R package version 2.7. 2015. Available: http://cran.r-project.org. Accessed on 2015-09-10.
184. Wu, Z. Y., Sun, H., Zhou, Z. K., Li, D. Z., Peng, H. Floristics of Seed Plants from China. Science Press, Beijing, China, pp. 52-108 (2010).
185. Zhang, R. Z. Zoogeography of China. Science Press, Beijing, China, pp. 487 (2011).

Table S1 Correlations between environmental variables in whole China and its three regions

Table S1-1 Whole China

Variables	1	2	3	4	5	6	7	8	9	10	11	12	13	14	15	16	17	18
2. Precipitation of the wettest quarter	0.96**																	
3. Precipitation of the driest quarter	0.92**	0.83**																
4. Mean annual dryness	0.96**	0.91**	0.88**															
5. Mean annual temperature	0.85**	0.82**	0.83**	0.72**														
6. Maximum temperature of the warmest month	0.43**	0.36**	0.57**	0.29**	0.69**													
7. Minimum temperature of the coldest month	0.87**	0.85**	0.81**	0.76**	0.97**	0.53**												
8. Annual potential evapotranspiration	0.53**	0.55**	0.51**	0.31**	0.79**	0.70**	0.72**											
9. Annual actual evapotranspiration	0.92**	0.88**	0.86**	0.86**	0.83**	0.44**	0.85**	0.56**										
10. Net primary productivity	0.83**	0.84**	0.72**	0.78**	0.73**	0.22**	0.80**	0.49**	0.78**									
11.Normalized difference vegetation index	0.46**	0.43**	0.42**	0.51**	0.21**	0.15**	0.19**	0.03	0.37**	0.44**								
12.Mean diurnal range	-0.82**	-0.73**	-0.83**	-0.78**	-0.80**	-0.51**	-0.83**	-0.36**	-0.74**	-0.67**	-0.32**							
13.Temperature seasonality	-0.70**	-0.70**	-0.54**	-0.66**	-0.64**	-0.02	-0.78**	-0.42**	-0.70**	-0.72**	-0.03	0.52**						
14.Temperature annual range	-0.81**	-0.80**	-0.68**	-0.76**	-0.78**	-0.18**	-0.90**	-0.51**	-0.80**	-0.80**	-0.10**	0.71**	0.96**					
15.Precipitation seasonality	-0.70**	-0.53**	-0.82**	-0.70**	-0.64**	-0.44**	-0.64**	-0.32**	-0.67**	-0.52**	-0.28**	0.70**	0.45**	0.55**				
16.Elevational range	0.05*	0.06**	-0.08**	0.13**	-0.17**	-0.54**	-0.03	-0.19**	0.03	0.14**	0.03	0.18**	-0.38**	-0.24**	-0.11**			
17.Mean elevation	-0.27**	-0.24**	-0.41**	-0.18**	-0.43**	-0.74**	-0.26**	-0.34**	-0.24**	-0.07**	-0.18**	0.44**	-0.24**	-0.04*	0.15**	0.79**		
18.Main land cover type	-0.38**	-0.35**	-0.30**	-0.44**	-0.16**	0.11**	-0.23**	-0.04*	-0.31**	-0.36**	-0.35**	0.17**	0.33**	0.30**	0.33**	-0.41**	-0.27**	
19.Number of land cover types	-0.15**	-0.12**	-0.20**	-0.08**	-0.24**	-0.38**	-0.20**	-0.24**	-0.16**	-0.13**	-0.21**	0.27**	-0.02	0.06**	0.06**	0.48**	0.38**	-0.14**

Note: the number in the first row corresponds to the number of variables in the first column. 1-Mean annual precipitation. Spearman (two-sided) correlation was performed (n=2376; **, p<0.01; *p<0.05).

Table S1-2 Eastern Monsoon Region of China

Variables	1	2	3	4	5	6	7	8	9	10	11	12	13	14	15	16	17	18
2. Precipitation of the wettest quarter	0.94**																	
3. Precipitation of the driest quarter	0.90**	0.77**																
4. Mean annual dryness	0.96**	0.89**	0.88**															
5. Mean annual temperature	0.83**	0.78**	0.79**	0.70**														
6. Maximum temperature of the warmest month	0.31**	0.20**	0.50**	0.19**	0.58**													
7. Minimum temperature of the coldest month	0.86**	0.83**	0.76**	0.75**	0.96**	0.38**												
8. Annual potential evapotranspiration	0.42**	0.46**	0.38**	0.19**	0.70**	0.57**	0.61**											
9. Annual actual evapotranspiration	0.90**	0.83**	0.83**	0.83**	0.81**	0.34**	0.83**	0.48**										
10. Net primary productivity	0.75**	0.77**	0.61**	0.70**	0.66**	0.02	0.77**	0.39**	0.71**									
11.Normalized difference vegetation index	0.27**	0.22**	0.24**	0.36**	-0.01	-0.03	-0.01	-0.21**	0.16**	0.22**								
12.Mean diurnal range	-0.74**	-0.63**	-0.76**	-0.76**	-0.72**	-0.37**	-0.78**	-0.12**	-0.67**	-0.54**	-0.10**							
13.Temperature seasonality	-0.78**	-0.78**	-0.59**	-0.69**	-0.77**	-0.03	-0.90**	-0.49**	-0.75**	-0.82**	0.06	0.62**						
14.Temperature annual range	-0.81**	-0.81**	-0.64**	-0.72**	-0.81**	-0.09**	-0.93**	-0.49**	-0.78**	-0.82**	0.06**	0.71**	0.99**					
15.Precipitation seasonality	-0.76**	-0.53**	-0.89**	-0.76**	-0.64**	-0.41**	-0.64**	-0.20**	-0.73**	-0.50**	-0.22**	0.73**	0.54**	0.55**				
16.Elevational range	0.34**	0.35**	0.15**	0.37**	0.07**	-0.45**	0.23**	0.01	0.29**	0.45**	0.27**	-0.03	-0.48**	-0.40**	-0.21**			
17.Mean elevation	0.03	0.07**	-0.17**	0.07**	-0.17**	-0.66**	0.02	-0.11**	0.040	0.31**	0.11**	0.19**	-0.36**	-0.24**	0.02	0.79**		
18.Main land cover type	-0.47**	-0.44**	-0.39**	-0.52**	-0.23**	0.10**	-0.31**	-0.07**	-0.40**	-0.43**	-0.41**	0.24**	0.38**	0.36**	0.36**	-0.47**	-0.32**	
19.Number of land cover types	0.16**	0.20**	0.06*	0.20**	0.01	-0.29**	0.07**	-0.06**	0.12**	0.16**	0.05*	0.04	-0.17**	-0.15**	-0.051*	0.41**	0.23**	-0.18**

Note: the number in the first row corresponds to the number of variables in the first column. 1-Mean annual precipitation. Spearman (two-sided) correlation was performed (n=1995; **, $p<0.01$; *$p<0.05$).

Table S1-3 Northwestern Arid Region of China

**Variables**	1	2	3	4	5	6	7	8	9	10	11	12	13	14	15	16	17	18
2. Precipitation of the wettest quarter	0.98**																	
3. Precipitation of the driest quarter	0.58**	0.46**																
4. Mean annual dryness	0.96**	0.94**	0.64**															
5. Mean annual temperature	-0.48**	-0.46**	-0.56**	-0.64**														
6. Maximum temperature of the warmest month	-0.55**	-0.52**	-0.53**	-0.68**	0.88**													
7. Minimum temperature of the coldest month	-0.45**	-0.44**	-0.54**	-0.60**	0.94**	0.69**												
8. Annual potential evapotranspiration	-0.59**	-0.57**	-0.65**	-0.74**	0.96**	0.87**	0.90**											
9. Annual actual evapotranspiration	0.92**	0.93**	0.51**	0.86**	-0.40**	-0.44**	-0.39**	-0.51**										
10. Net primary productivity	0.86**	0.83**	0.59**	0.87**	-0.46**	-0.52**	-0.43**	-0.60**	0.78**									
11.Normalized difference vegetation index	0.77**	0.74**	0.59**	0.82**	-0.52**	-0.51**	-0.50**	-0.64**	0.71**	0.93**								
12.Mean diurnal range	-0.47**	-0.40**	-0.68**	-0.54**	0.41**	0.42**	0.36**	0.58**	-0.45**	-0.53**	-0.53**							
13.Temperature seasonality	0.25**	0.27**	0.35**	0.31**	-0.44**	-0.04	-0.66**	-0.47**	0.30**	0.27**	0.37**	-0.29**						
14.Temperature annual range	-0.01	0.04	0.06	0.02	-0.21**	0.21**	-0.48**	-0.17*	0.08	-0.01	0.10	0.10	0.89**					
15.Precipitation seasonality	0.59**	0.72**	-0.14*	0.53**	-0.26**	-0.22**	-0.30**	-0.27**	0.63**	0.43**	0.40**	0.03	0.26**	0.24**				
16.Elevational range	-0.33**	-0.39**	-0.09	-0.22**	-0.14*	-0.17*	-0.09	-0.06	-0.40**	-0.29**	-0.26**	-0.04	-0.14*	-0.16*	-0.52**			
17.Mean elevation	-0.24**	-0.27**	-0.07	-0.17*	-0.14*	-0.42**	0.09	-0.06	-0.31**	-0.25**	-0.30**	0.02	-0.65**	-0.67**	-0.32**	0.36**		
18.Main land cover type	-0.23**	-0.18**	-0.38**	-0.27**	0.42**	0.42**	0.33**	0.43**	-0.17*	-0.19**	-0.15*	0.27**	-0.04	0.08	0.08	-0.05	-0.26**	
19.Number of land cover types	-0.45**	-0.46**	-0.19**	-0.39**	0.08	0.14*	0.05	0.17*	-0.42**	-0.50**	-0.49**	0.15*	-0.07	0.03	-0.35**	0.60**	0.19**	0.07

Note: the number in the first row corresponds to the number of variables in the first column. 1-Mean annual precipitation. Spearman (two-sided) correlation was performed (n=210; **, p<0.01; *p<0.05).

Table S1-4 Qinghai-Tibetan Plateau Region of China

Variables	1	2	3	4	5	6	7	8	9	10	11	12	13	14	15	16	17	18
2. Precipitation of the wettest quarter	0.98**																	
3. Precipitation of the driest quarter	0.77**	0.71**																
4. Mean annual dryness	0.96**	0.93**	0.82**															
5. Mean annual temperature	0.55**	0.56**	0.22**	0.34**														
6. Maximum temperature of the warmest month	0.25**	0.23**	0.05	0.08	0.78**													
7. Minimum temperature of the coldest month	0.58**	0.61**	0.37**	0.41**	0.87**	0.46**												
8. Annual potential evapotranspiration	0.44**	0.46**	0.11	0.22**	0.97**	0.79**	0.81**											
9. Annual actual evapotranspiration	0.73**	0.74**	0.70**	0.68**	0.42**	0.01	0.62**	0.36**										
10. Net primary productivity	0.87**	0.84**	0.53**	0.78**	0.71**	0.46**	0.62**	0.60**	0.58**									
11.Normalized difference vegetation index	0.79**	0.75**	0.49**	0.76**	0.41**	0.33**	0.25**	0.32**	0.39**	0.84**								
12.Mean diurnal range	-0.30**	-0.26**	-0.47**	-0.35**	-0.19*	-0.13	-0.36**	-0.01	-0.21**	-0.25**	-0.07							
13.Temperature seasonality	-0.52**	-0.57**	-0.38**	-0.43**	-0.49**	0.09	-0.77**	-0.44**	-0.76**	-0.46**	-0.12	0.13						
14.Temperature annual range	-0.50**	-0.55**	-0.43**	-0.43**	-0.44**	0.10	-0.79**	-0.38**	-0.74**	-0.41**	-0.05	0.33**	0.94**					
15.Precipitation seasonality	-0.55**	-0.44**	-0.74**	-0.58**	-0.27**	-0.40**	-0.19*	-0.16*	-0.27**	-0.47*	-0.51**	0.49**	-0.06	-0.05				
16. Elevational range	0.17*	0.18*	0.25**	0.15*	0.25**	0.12	0.38**	0.20*	0.29**	0.14	-0.22**	-0.44**	-0.40**	-0.44**	-0.18*			
17. Mean elevation	-0.48**	-0.42**	-0.28**	-0.40**	-0.66**	-0.84**	-0.34**	-0.59**	-0.10	-0.65**	-0.59**	0.31**	-0.15	-0.15*	0.60**	-0.11		
18.Main land cover type	-0.60**	-0.58**	-0.33**	-0.52**	-0.51**	-0.38**	-0.40**	-0.45**	-0.33**	-0.64**	-0.61**	0.13	0.25**	0.17*	0.38**	-0.07	0.47**	
19.Number of land cover types	-0.40**	-0.40**	-0.19*	-0.33**	-0.30**	-0.19*	-0.20**	-0.33**	-0.24**	-0.40**	-0.56**	-0.23**	0.12	0.07	0.15	0.45**	0.18*	0.39**

Note: the number in the first row corresponds to the number of variables in the first column. 1-Mean annual precipitation. Spearman (two-sided) correlation was performed (n=171; **, p<0.01; *p<0.05).

Table S2 Variables selected based on Spearman (two-sided) correlation analysis and univariate regression for the residuals of species richness of vascular plants and mammals in whole China and its three regions. Spearman (two-sided) correlation analysis between any two variables in each category was performed. The deviance of each variable in each category explaining species richness was calculated using univariate regression models. If the correlation coefficient between two variables in a category was >0.7, we identified strongly intercorrelated variables and retained the variables that explained more deviance in univariate regression models. Mean annual precipitation: PRE; precipitation of the wettest quarter: PREmax; precipitation of the driest quarter: PREmin; mean annual dryness: DRY; mean annual temperature: TEM; maximum temperature of the warmest month: TEMmax; minimum temperature of the coldest month: TEMmin; annual potential evapotranspiration: PET; annual actual evapotranspiration: AET; net primary productivity: NPP; temperature annual range: TEMvar; mean diurnal range: TEMday; temperature seasonality: TEMsea; precipitation seasonality: PREsea; elevational range: ELEV; mean elevation: ELEM; main land cover type: LC.

Table S2-1 Vascular plants in whole China

Hypotheses and variables	Deviance (%)
**Water**	
PRE	23.7
**Ambient energy (temperature)**	
TEMmax	1.6
TEMmin	9.9
**Productivity**	
NPP	22.3
**Environmental stability**	
TEMsea	4.2
PREsea	4.1
TEMday	12.6
**Habitat heterogeneity**	
ELEV	13.8
LC	5.2
Number of land cover types	1.9

Table S2-2 Vascular plants in Eastern Monsoon Region of China

Hypotheses and variables	Deviance (%)
**Water**	
PRE	18.0
**Ambient energy (temperature)**	
TEMmax	0.6
TEMmin	16.3
PET	0.3

**Productivity**	
AET	9.5
**Environmental stability**	
TEMvar	15.4
PREsea	4.3
**Habitat heterogeneity**	
ELEV	22.8
LC	1.6
Number of land cover types	0.4

Table S2-3 Vascular plants in Northwestern Arid Region of China

Hypotheses and variables	Deviance (%)
**Water**	
PREmin	19.4
**Ambient energy (temperature)**	
TEMmax	11.3
**Productivity**	
NDVI	6.5
**Environmental stability**	
TEMday	6.0
PREsea	5.8
**Habitat heterogeneity**	
ELEV	20.5
LC	6.3
Number of land cover types	4.5

Table S2-4 Vascular plants in Qinghai-Tibet Plateau Region of China

Hypotheses and variables	Deviance (%)
**Water**	
PREmin	31.2
**Ambient energy (temperature)**	
TEMmin	9.5
**Productivity**	
NPP	21.8
**Environmental stability**	
TEMday	12.7
TEMvar	1.4
PREsea	24.2
**Habitat heterogeneity**	
ELEV	11.9
Number of land cover types	9.9
LC	5.9

Table S2-5 Mammals in whole China

Hypotheses and variables	Deviance (%)
**Water**	
PRE	16.4
**Ambient energy (temperature)**	
TEMmax	0.1
TEMmin	9.4
**Productivity**	
NPP	17.2
**Environmental stability**	
TEMvar	12.4
PREsea	2.1
**Habitat heterogeneity**	
ELEV	10.4
LC	6.4
Number of land cover types	1.1

Table S2-6 Mammals in Eastern Monsoon Region of China

Hypotheses and variables	Deviance (%)
**Water**	
PRE	15.2
**Ambient energy (temperature)**	
TEMmax	0.7
TEMmin	12.0
**Productivity**	
NPP	10.8
**Environmental stability**	
TEMsea	12.6
PREsea	1.5
**Habitat heterogeneity**	
ELEV	11.6
LC	3.0
Number of land cover types	0.3

Table S2-7 Mammals in Northwestern Arid Region of China

Hypotheses and variables	Deviance (%)
**Water**	
PREmax	1.2
PREmin	19.1
**Ambient energy (temperature)**	
TEMmax	3.7

**Productivity**	
NDVI	1.0
**Environmental stability**	
PREsea	16.9
**Habitat heterogeneity**	
ELEV	13.3
Number of land cover types	8.7

Table S2-8 Mammals in Qinghai-Tibet Plateau Region of China

Hypotheses and variables	Deviance (%)
**Water**	
DRY	37.0
**Ambient energy (temperature)**	
TEMmin	11.7
**Productivity**	
NPP	28.8
**Environmental stability**	
TEMvar	10.3
PREsea	32.7
**Habitat heterogeneity**	
ELEV	10.9
ELEM	9.8
LC	14.4

Table S3 Top six predictors selected based on the percentage of a variable reaching statistical significance among 1000 replicates. We established GLM models with the selected predictors (see Table S2), and calculated the statistical significance at $p<0.05$ for the regression coefficient of each variable. This process was replicated 1000 times with randomly generated samples. We summed the number of times regression coefficient of each variable reached statistical significance. Top six variables were selected according to the percentage of a variable reaching statistical significance among 1000 times. Mean annual precipitation: PRE; precipitation of the wettest quarter: PREmax; precipitation of the driest quarter: PREmin; mean annual dryness: DRY; mean annual temperature: TEM; maximum temperature of the warmest month: TEMmax; minimum temperature of the coldest month: TEMmin; annual potential evapotranspiration: PET; net primary productivity: NPP; normalized difference vegetation index: NDVI; temperature annual range: TEMvar; mean diurnal range: TEMday; temperature seasonality: TEMsea; precipitation seasonality: PREsea; elevational range: ELEV; mean elevation: ELEM; main land cover type: LC.

No.	Predictors	Percentage of a variable reached statistical significance among 1000 times
	**Vascular plants in whole China**	
1	ELEV	100
2	PREsea	100
3	NPP	100
4	PRE	99.50
5	TEMmax	98.87
6	LC	92.79
	**Vascular plants in Eastern Monsoon Region**	
1	ELEV	100
2	PREsea	100
3	TEMmax	99.50
4	TEMmin	94.55
5	LC	87.59
6	PET	82.38
	**Vascular plants in Northwestern Arid Region**	
1	ELEV	100
2	NDVI	100
3	PREsea	100
4	LC	79.19
5	PREmin	61.84
6	TEMday	48.07
	**Vascular plants in Qinghai-Tibet Plateau Region**	
1	NPP	99.19
2	ELEV	94.49

3	PREsea	92.38
4	PREmin	79.84
5	TEMmin	77.72
6	TEMvar	75.71
	**Mammals in whole China**	
1	ELEV	100
2	NPP	100
3	LC	100
4	TEMmax	85.22
5	PREsea	84.41
6	TEMvar	83.68
	**Mammals in Eastern Monsoon Region**	
1	ELEV	100
2	PRE	100
3	TEMmax	100
4	NPP	99.40
5	LC	98.59
6	PREsea	76.36
	**Mammals in Northwestern Arid Region**	
1	ELEV	93.40
2	PREmin	92.55
3	PREmax	89.79
4	PREsea	88.86
5	NDVI	81.66
6	Number of land cover types	64.34
	**Mammals in Qinghai-Tibet Plateau Region**	
1	ELEV	98.77
2	DRY	94.23
3	PREsea	90.92
4	NPP	87.75
5	ELEM	84.70
6	TEMmin	76.32

Table S4 Top six predictors selected based on stratified random sampling. We selected subsets of samples (60%, 70%, 80% and 90%) from the target regions (i.e., whole China, EMR, NAR and QTR respectively) using stratified random sampling with a bootstrap procedure, and compared the multivariate models based on the subsets of samples with that of target regions (100%). When p<0.05, the regression coefficient is considered statistically significant. This process was replicated 1000 times with randomly generated samples. We counted the number of times the regression coefficient of each variable reached statistical significance. The top six variables were selected according to the number of times each variable reached statistical significance among 1000 times. Mean annual precipitation: PRE; precipitation of the wettest quarter: PREmax; precipitation of the driest quarter: PREmin; mean annual dryness: DRY; mean annual temperature: TEM; maximum temperature of the warmest month: TEMmax; minimum temperature of the coldest month: TEMmin; annual potential evapotranspiration: PET; net primary productivity: NPP; normalized difference vegetation index: NDVI; temperature annual range: TEMvar; mean diurnal range: TEMday; temperature seasonality: TEMsea; precipitation seasonality: PREsea; elevational range: ELEV; mean elevation: ELEM; main land cover type: LC. Data in parentheses are the percentage of a variable that reached statistical significance at p<0.05 among 1000 times.

**No.**	**Sampling proportion 60%**	**Sampling proportion 70%**	**Sampling proportion 80%**	**Sampling proportion 90%**	**Target region 100%**
	**Vascular plants in whole China**				
1	ELEV (100)	ELEV (100)	ELEV (100)	ELEV (100)	ELEV (100)
2	NPP (100)	PREsea (100)	PREsea (100)	PREsea (100)	PREsea (100)
3	PREsea (99.90)	NPP (100)	NPP (100)	NPP (100)	NPP (100)
4	TEMmax (99.60)	TEMmax (100)	PRE (100)	PRE (100)	PRE (99.50)
5	PRE (98.39)	PRE (99.50)	TEMmax (100)	TEMmax (100)	TEMmax (98.87)
6	LC (87.50)	LC (93.33)	LC (98.70)	LC (100)	LC (92.79)
	**Vascular plants in Eastern Monsoon Region**				
1	ELEV (100)	ELEV (100)	ELEV (100)	ELEV (100)	ELEV (100)
2	PREsea (100)	PREsea (100)	PREsea (100)	PREsea (100)	PREsea (100)
3	TEMmax (99.40)	TEMmax (99.90)	TEMmax (100)	TEMmax (100)	TEMmax (99.50)
4	TEMmin (91.66)	TEMmin (94.17)	TEMmin (97.70)	TEMmin (100)	TEMmin (94.55)
5	LC (76.13)	LC (75.91)	LC (84.07)	LC (95.10	LC (87.59)
6	PRE (58.22)	PRE (61.78)	PET (65.49)	PET (79.88)	PET (82.38)
	**Vascular plants in Northwestern Arid Region**				
1	ELEV (100)	ELEV (100)	ELEV (100)	ELEV (100)	ELEV (100)
2	NDVI (98.59)	NDVI (97.05)	NDVI (99.80)	NDVI (100)	NDVI (100)
3	PREsea (84.25)	PREsea (85.22)	PREsea (98.89)	PREsea (100)	PREsea (100)
4	LC (55.97)	LC (53.21)	PREmin (77.78)	PREmin (100)	LC (79.19)
5	PREmin (52.69)	PREmin (52.79)	LC (62.47)	LC (79.19)	PREmin (61.84)
6	TEMday (40.10)	TEMday (42.11)	TEMday (44.46)	TEMday (48.07)	TEMday (48.07)

	**Vascular plants in Qinghai-Tibet Plateau Region**				
1	NPP (98.29)	NPP (99.50)	NPP (100)	NPP (100)	NPP (99.19)
2	ELEV (93.23)	ELEV (96.73)	ELEV (98.59)	ELEV (99.80)	ELEV (94.49)
3	PREsea (90.40)	PREsea (93.17)	PREsea (95.03)	PREsea (98.70)	PREsea (92.38)
4	TEMmin (65.07)	TEMmin (67.97)	TEMmin (70.02)	TEMmin (77.79)	PREmin (79.84)
5	TEMvar (58.74)	TEMvar (53.93)	TEMvar (51.37)	TEMvar (56.04)	TEMmin (77.72)
6	PREmin (52.35)	PREmin (48.78)	PREmin (48.43)	PREmin (53.69)	TEMvar (75.71)
	**Mammals in whole China**				
1	ELEV (100)	ELEV (100)	ELEV (100)	ELEV (100)	ELEV (100)
2	NPP (100)	NPP (100)	NPP (100)	NPP (100)	NPP (100)
3	LC (100)	LC (100)	LC (100)	LC (100)	LC (100)
4	PREsea (73.54)	TEMvar (79.27)	PREsea (83.63)	PREsea (95.60)	TEMmax (85.22)
5	TEMvar (72.81)	PREsea (77.87)	TEMmax (81.48)	TEMvar (93.66)	PREsea (84.41)
6	TEMmax (71.50)	TEMmax (77.80)	TEMvar (81.22)	TEMmax (92.20)	TEMvar (83.68)
	**Mammals in Eastern Monsoon Region**				
1	ELEV (100)	ELEV (100)	ELEV (100)	ELEV (100)	ELEV (100)
2	PRE (100)	NPP (100)	NPP (100)	NPP (100)	PRE (100)
3	TEMmax (100)	PRE (100)	PRE (100)	PRE (100)	TEMmax (100)
4	NPP (99.40)	TEMmax (100)	LC (100)	LC (100)	NPP (99.40)
5	LC (97.99)	LC (99.00)	TEMmax (100)	TEMmax (100)	LC (98.59)
6	PREsea (49.11)	PREsea (46.16)	PREsea (46.13)	PREsea (42.44)	PREsea (76.36)
	**Mammals in Northwestern Arid Region**				
1	ELEV (89.12)	PREsea (92.42)	PREmin (98.67)	PREsea (100)	ELEV (93.40)
2	PREsea (87.80)	PREmin (90.20)	PREsea (97.98)	PREmin (99.85)	PREmin (92.55)
3	PREmax (86.57)	ELEV (89.43)	ELEV (95.34)	ELEV (99.30)	PREmax (89.79)
4	PREmin (84.19)	PREmax (87.91)	PREmax (84.16)	NDVI (86.83)	PREsea (88.86)
5	NDVI (75.18)	NDVI (75.13)	NDVI (78.45)	PREmax (80.20)	NDVI (81.66)
6	TEMmax (51.33)	TEMmax (40.92)	TEMmax (32.08)	TEMmax (19.34)	Number of land cover types (64.34)
	**Mammals in Qinghai-Tibet Plateau Region**				
1	ELEV (96.85)	ELEV (98.69)	ELEV (99.80)	ELEV (100)	ELEV (98.77)
2	PREsea (85.70)	PREsea (92.88)	PREsea (97.57)	PREsea (99.70)	DRY (94.23)
3	NPP (82.43)	NPP (83.17)	DRY (91.55)	DRY (97.80)	PREsea (90.92)
4	DRY (80.00)	DRY (80.94)	NPP (88.42)	NPP (95.39)	NPP (87.75)
5	ELEM (70.06)	ELEM (66.48)	ELEM (67.30)	ELEM (76.85)	ELEM (84.70)
6	TEMvar (64.75)	TEMvar (57.58)	TEMmin (61.94)	TEMmin (52.63)	TEMmin (76.32)

Table S5 SLM multivariate models for species richness of vascular plants across China and its three regions with area as a variable in the models. We divided the area of China into three regions: Eastern Monsoon Region (EMR, n=1995), Northwestern Arid Region (NAR, n=210), and Qinghai-Tibet Plateau Region (QTR, n=171). Area of counties was used as a variable. Species richness, areas and environmental variables were log_{10}-transformed. Data in parentheses are the results of new methods which include area as an environmental variable and raw data of species richness were used (*Pr(>|z|)<0.05, ** Pr(>|z|)<0.01, *** Pr(>|z|)<0.001).

Model	Predictors		Whole China	EMR	NAR	QTR
Model with 6 predictors	Elevational range	z	15.68*** (13.75***)	9.19*** (13.04***)	6.34*** (6.28***)	4.15*** (3.45***)
	Net primary productivity	z	4.83*** (5.09***)	-	-	5.59*** (6.63***)
	Normalized difference vegetation index	z	-	-	5.47*** (3.43***)	-
	Maximum temperature of the warmest month	z	3.64***	-2.72** (-4.41***)	-	-
	Minimum temperature of the coldest month	z	-	6.22***	-	-1.72
	Annual potential evapotranspiration	z	-	-1.95 (2.22*)	-	-
	Mean diurnal range	z	-	-	0.85 (1.30)	- (0.39)
	Temperature annual range	z	- (1.09)	-	-	0.03 (0.50)
	Temperature seasonality	z	- (-0.14)	-	-	-
	Mean annual precipitation	z	6.80*** (7.20***)	-	-	-
	Precipitation of the driest quarter	z	-	-	-0.33 (0.02)	1.37 (0.90)
	Mean annual dryness	z	-	- (4.82***)	-	-
	Precipitation seasonality	z	-3.83*** (-5.78***)	-2.35* (-1.84)	-1.79 (-2.07*)	-2.18* (-2.34*)
	Main land cover type	z	-4.11***	-2.23* (-2.29*)	-1.48 (-2.01*)	-
	Number of land cover types	z	-	-	- (-0.28)	-
	Area	z	(4.28***)	(5.39***)	-	(2.77**)
	AIC		-1147.1 (-1127.6)	-1606.7 (-1839.2)	-147.5 (-143.1)	153.6 (144.1)
	Fitted values	r^2	0.61 (0.61)	0.58 (0.68)	0.53 (0.53)	0.51 (0.54)
	Moran's I		-0.06 (-0.06)	-0.02 (-0.01)	-0.08 (-0.02)	0.00 (0.00)
19(20)-predictor model	AIC		-1202.6 (-1198.7)	-1626.8 (-1859.1)	-148.7 (-147.1)	167.4 (161.1)
	Fitted values	r^2	0.62 (0.63)	0.60 (0.69)	0.58 (0.60)	0.55 (0.57)

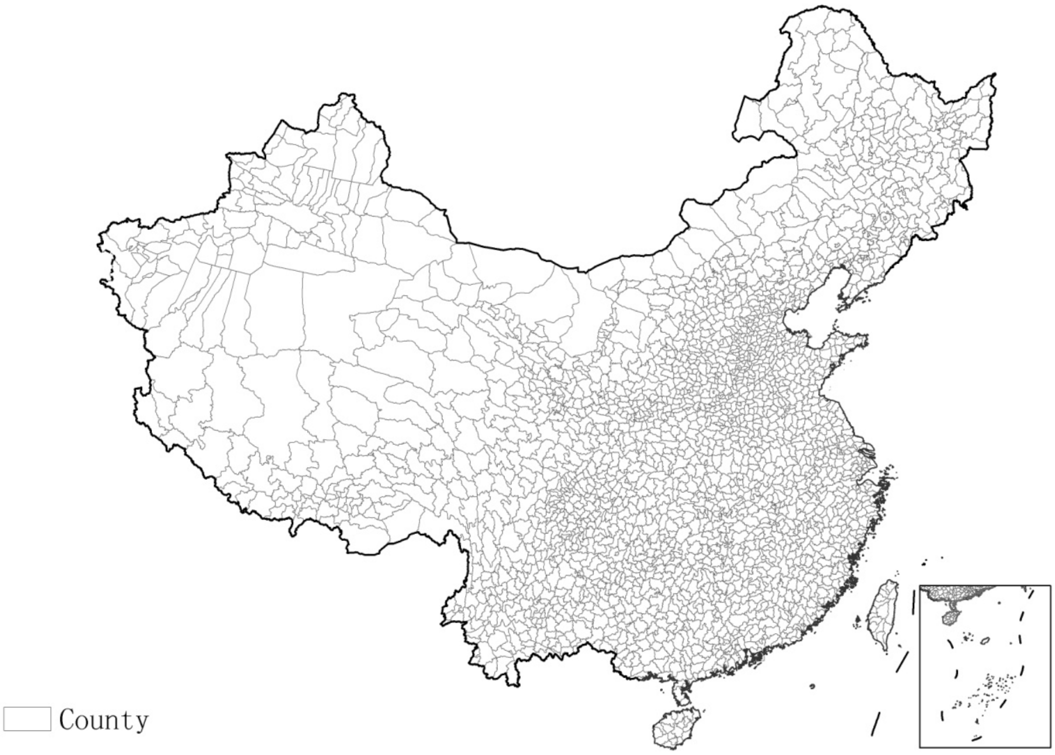

Fig. S1 Map of assessment units in China. Marine ecosystems were not included in this study. Data on the boundary of assessment units were from the National Administration of Surveying, Mapping and Geoinformation of China (http://www.sbsm.gov.cn/). The map was created using the software Arc GIS 9.3 which was purchased from ESRI-China (http://www.esrichina-bj.cn/) by Nanjing Institute of Environmental Sciences affiliated to the Ministry of Environmental Protection of China, with user number C-801668.
